# Heat Stress Responsive Aux/IAA Protein, OsIAA29 Regulates Grain Filling Through OsARF17 Mediated Auxin Signaling Pathway

**DOI:** 10.1186/s12284-024-00694-z

**Published:** 2024-02-19

**Authors:** Zhanghao Chen, Wei Zhou, Xianyu Guo, Sheng Ling, Wang Li, Xin Wang, Jialing Yao

**Affiliations:** 1 College of Life Science and Technology, Huazhong Agricultural University, Wuhan, 430070 China; 2 Key Laboratory of Molecular Biology and Genetic Engineering of Jiangxi Province, School of Life Sciences, Nanchang University, Nanchang, 330031 China; 3 College of Life Sciences, Xinyang Normal University, Xinyang, 464000 China

**Keywords:** *Oryza sativa*, AUX, IAA, *OsIAA29*, Seed specific expression, Heat stress, Seed development

## Abstract

High temperature during grain filling considerably reduces yield and quality in rice, but its molecular mechanisms are not fully understood. We investigated the functions of a seed preferentially expressed Aux/IAA gene, *OsIAA29*, under high temperature-stress in grain filling using CRISPR/Cas9, RNAi, and overexpression. We observed that the *osiaa29* had a higher percentage of shrunken and chalkiness seed, as well as lower 1000-grain weight than ZH11 under high temperature. Meanwhile, the expression of *OsIAA29* was induced and the IAA content was remarkably reduced in the ZH11 seeds under high temperature. In addition, OsIAA29 may enhance the transcriptional activation activity of OsARF17 through competition with OsIAA21 binding to OsARF17. Finally, chromatin immunoprecipitation quantitative real-time PCR (ChIP-qPCR) results proved that OsARF17 regulated expression of several starch and protein synthesis related genes (like *OsPDIL1-1*, *OsSS1*, *OsNAC20*, *OsSBE1*, and *OsC2H2*). Therefore, OsIAA29 regulates seed development in high temperature through competition with OsIAA21 in the binding to OsARF17, mediating auxin signaling pathway in rice. This study provides a theoretical basis and gene resources for auxin signaling and effective molecular design breeding.

**Supplementary Information:**

The online version contains supplementary material available at 10.1186/s12284-024-00694-z.

## Introduction

Rice (*Oryza sativa*) feeds more than half of the world's population. Recently, as the greenhouse effect causes global climate warming, high temperatures frequent occurrence in rice producing areas including South Asia, East Asia, Southeast Asia, and China. In general, when the daily mean temperatures are higher than 30 °C constantly or the daytime temperatures exceed 35 °C, heat damage to rice will be inevitable. The high temperature sensitive period in rice includes the booting stage, heading stage, and filling stage. The heat damage resulted in a significant and irreversible reduction in rice grain production by affecting seed setting rate, thousand-grain weight, and grain filling. It was reported that an average of 1 °C increase in temperature would lead to approximately 3.2% reduction in rice yield (Peng et al. 2004; Xu et al. 2020). High temperature also severely affects rice grain quality through impairing synthesis, metabolism, and transport of the major storage substances (Nevame et al. 2018).

Recently, a number of studies have been reported on the isolation and identification of responding heat stress in the booting stage and heading stage of rice. For example, *qHd1* confers tolerance to high temperature at the heading and grain-filling stages, under natural high temperature conditions, the *qHd1* allele genotype ZS97 has higher yields than allele genotype MY46 (Chen et al. 2018). OsFIE1, a member of Polycomb Repressive Complex2 family, was demethylated by heat stress to negatively regulate seed development (Dhatt et al. 2021). OsProDH was a thermotolerance regulator that affected tolerance to heat stress through modulating proline metabolism and scavenging reactive oxygen species (Guo et al. 2020). In our previous study, we found that the NAC transcription factors ONAC127 and ONAC129 could form a heterodimer and be involved in high temperatures responses, which played significant roles in rice grain filling (Ren et al. 2021). Auxins signaling pathway is known to be engaged in the regulation of heat stress response (Ai et al. 2023).

The auxin/indole-3-acetic acid (Aux/IAA) and auxin response factor (ARF) genes, two crucial gene families, play important roles in the plant growth and development through regulating auxin signaling pathway (Ramos Baez and Nemhauser 2021). Such as, OsIAA10-OsARF4 model regulates grain size through auxin signaling (Ma et al. 2023b). OsSK41-OsIAA10-OsARF regulates grain yield traits via auxin signaling in rice (Ma et al. 2023a). OsSGS3-tasiRNA-OsARF3 module regulates distinct immunity and thermotolerance (Gu et al. 2023). Some Aux/IAAs lack the conserved domain I and II, which are called Non-canonical AUX/IAA. Recent research exposed that the non-canonical AUX/IAA proteins were essential for the auxin to control plant growth and development (Lv et al. 2020). Currently, how the auxin signaling pathway regulates early reproductive development and heat-stress response in seeds remains to be an outstanding question.

The auxin-inducible Aux/IAA gene family comprises 31 members by screening the available rice databases, they were designated as OsIAA1 to 31. In our previous study, we identified that *OsIAA29* was highly expressed in seeds (Nie et al. 2013). In this study, we demonstrated that OsIAA29 was a Non-canonical AUX/IAA protein without typical domains I and II, which was induced by high temperature. OsIAA29 had higher expression in high temperature and its mutants with a more severe phenotype under high temperature. We found that OsIAA29 might regulate auxin signal pathway by competing with OsIAA21 forming a complex with OsARF17 to regulate rice grain filling when rice is exposed to high temperatures. These discoveries may improve the understanding of the auxin signaling pathways in response to heat stress in grain filling.

## Results

### Sequence Characteristics and Domain Analysis of OsIAA29

**Fig. 1:**
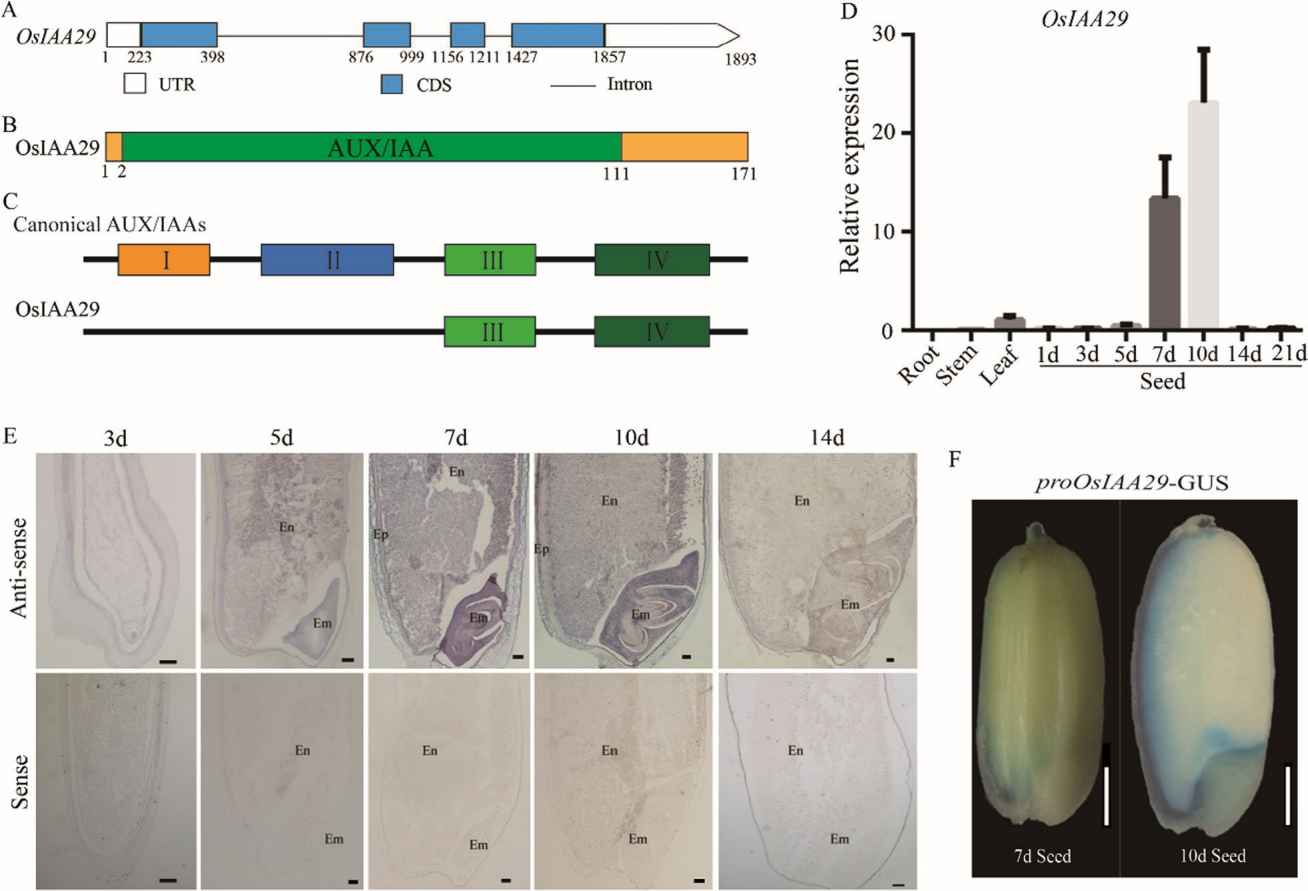
Sequence and expression pattern of OsIAA29. **A** The structure of OsIAA29. The blue boxes represent the exons, the black lines represent the intron and the white boxes the UTRs. **B** Schematic diagram of the domains of OsIAA29. **C** Compared with other canonical AUX/IAA, OsIAA29 lacks I and II domains. **D** qRT-PCR analysis of Os*IAA29* in different tissues. **E** In situ hybridization of sectioned caryopses collected at 3, 5, 7, 10, and 14 DAP (day after pollination) using antisense and sense probes. *OsIAA29* was highly expressed in the seed embryo layer and the starchy endosperm. Em, Embryo; Ep, endopleura; AL, Aleurone, scale bars are 100 µm. **F** Histochemical GUS activity staining using pro*OsIAA29*::GUS, scale bars are 1 mm

The genomic DNA (1893 bp) and CDS (516 bp) sequence of *OsIAA29* were obtained using the NCBI database (http://www.ncbi.nlm.nih.gov/). *OsIAA29* is composed of four exons and three introns by analyzing its full-length genomic sequence (Fig. 1A). The protein domain analysis confirmed OsIAA29 as an AUX/IAAs protein. Further analysis found that the subdomain I and II, which mediated the repression of ARF-transcription and facilitated the interaction between AUX/IAA protein with TIR1, were missing in OsIAA29 (Fig. 1B, C). This indicated that OsIAA29 might be a Non-canonical AUX/IAA protein.

### *OsIAA29 *was Highly Expressed in Developing Seed

To investigate the spatial and temporal expression pattern of *OsIAA29*, we isolated the total RNA of rice developing seeds (1-21 DAP, day after pollination) and vegetative tissues for qRT-PCR. The result showed *OsIAA29* was predominantly expressed in 7 DAP and 10 DAP seeds, which reached its maximum expression level at 10 DAP (Fig. 1D). In contrast, the expression levels of *OsIAA29* in other tested tissues were much lower.

To determine the spatial distribution of *OsIAA29* RNA, the mRNA in situ hybridization analysis was performed using the developing seeds of ZH11. It was found that *OsIAA29* was dominantly expressed in the embryo and endosperm of 7 DAP and 10 DAP seeds (Fig. 1E). The spatial expression pattern of *OsIAA29* was further examined by GUS (β-glucuronidase) using five *ProOsIAA29*::GUS transgenic plants. The results showed *OsIAA29* was predominantly expressed in the dorsal aleurone, directly under the vascular bundle and embryo and endosperm intermediate (Fig. 1F). Our results are similar to previous reports (Basunia et al. 2021). The expression pattern suggests that *OsIAA29* might play important roles in seed development of rice.

### Disruption of OsIAA29 Caused a Decrease in Grain Weight and Quality, Especially Under High Temperature

**Fig. 2:**
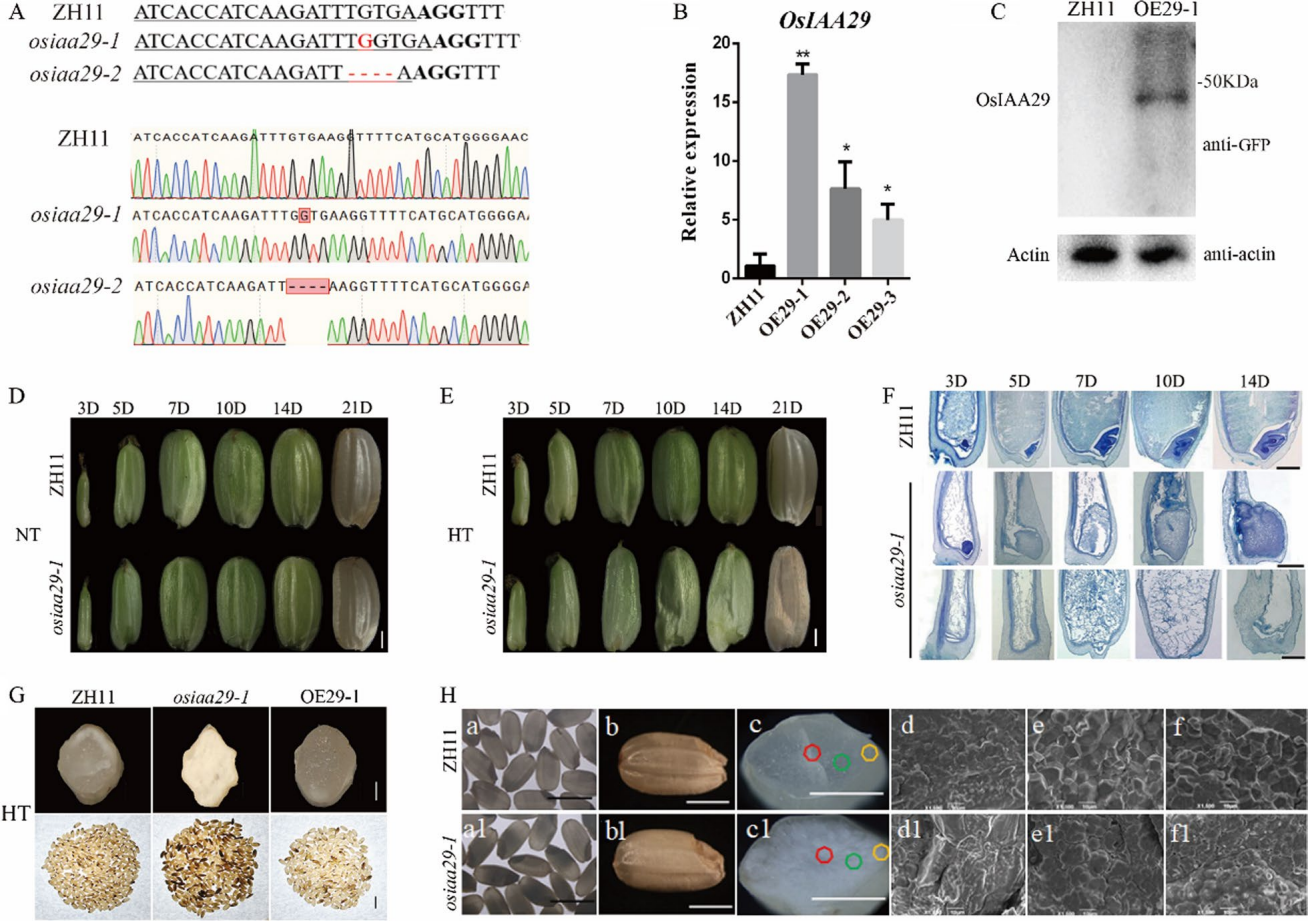
Phenotypic analysis of *OsIAA29* CRISPR lines (*osiaa29*) and overexpression plants (OE29). **A** Mutation sites in *osiaa29-1* and *osiaa29-2*, as compared with wild-type (WT) sequences, protospacer-adjacent motif sequences are shown in bold, and inserted or deleted nucleotides are indicated in red. **B** Relative expression level of overexpression materials lines in ZH11. Data are mean standard error (SE) for three replicates. *P < 0.05, **P < 0.01. *P*-values produced by two-tailed Student’s *t*-test. **C** Detection of eGFP fusion proteins in ZH11 and *OsIAA29* overexpression line (OE29-1). Total proteins extracted from developing caryopses at 7-DAP were used for western blot analysis with an anti-GFP antibody. **D** the appearance of *osiaa29-1* did not change under normal conditions. **E** the shrunken seeds *osiaa29-1* material in the filling stage under high temperature conditions. Seeds at 3, 5, 7, 10, 14, and 21 DAF were observed. Scale bars are 1 mm. **F** images of the seeds longitudinal section the in different periods and stained with toluidine blue. **G** The WT, *oaiaa29-1*, and OE29-1 images of 200 grains of mature seeds; scale bars are 10 mm. And cross-sections of the seeds; scale bars are 1 mm. **H** SEM images of the mature endosperm at different parts: the areas are indicated by the squares in (c-c1). Scale bars are 5 mm (a-a1); 2 mm (b-b1); 2 mm (c-c1); 10 μm (d-f1)

To explore the functions of *OsIAA29* in rice, we created CRISPR transgenic lines (*osiaa29*), RNAi lines (Ri29), and overexpression lines (OE29) of *OsIAA29*. We identified two types of mutants: *osiaa29-1* line with a 1-nt (+G) insertion, and *osiaa29-2* line with a 4-nt deletion (-TGTC) (Fig. 2A). Each of the predicted mutations produced earlier translation termination compared with WT, destroying of the AUX/IAA domain. The off-target detection was performed in the homologous lines of *osiaa29* and no off-target cleavage was found at either of the possible mutation sites (Additional file 1: Table S2). Two independent homozygous lines (*osiaa29-1* and *osiaa29-2*) were advanced up to T_4_ generation for analyzing the functions of *OsIAA29*. No T-DNA was detected in these transgenic lines by PCR analysis. The overexpression plants were further detected using quantitative reverse-transcription PCR (qRT-PCR). It was found that the expression level of *OsIAA29* was enhanced in OE29-1, OE29-2, and OE29-3 (Fig. 2B). Furthermore, western blot was performed to detect the expression of OsIAA29 in OE29-1 line. The results demonstrated that a 50 ~ KDa target protein was present in the total protein of OE29-1 line, while it was not detected in WT. (Fig. 2C). Three independent transgenic lines (OE29-1, OE29-2, and OE29-3) were advanced up to T_2_ generation for analyzing the functions of OsIAA29.

Previous researches showed that temperature above 35 °C at the flowering and grain filling stages has a huge negative impact on yield (Hakata et al. 2017). We performed high temperature stress assay using *osiaa29* lines in paddy field in 2018 summer. The results showed that 1000-grain weight of *osiaa29* lines were significantly reduced compare with WT, in addition to a higher rate of shrunken and higher chalkiness seed under high-temperature condition (Additional file 1: Table S3). Two batches of rice were cultivated in 2019, which flowered around 3 July 2019 and 5 August 2019, respectively. Given that OsIAA29 functions at the early grain filling stage, the heat damage accumulated during 0–7 DAP (T_S7_) and heat damage hours during 0–7 DAP (H_S7_) were calculated. Since the TS and HS of the second batch plants were significantly higher than those of the first batch, especially in early-stage of seed development (Additional file 1: Fig. S1), we defined the plants cultivated in the second batch as suffering from heat stress, while those planted in the first batch as growing under normal conditions with little or no heat stress. No morphological/phenotypic differences were observed in transgenic lines compared with WT until flowering stage. A significant reduction in 1000-grain weight was observed for *osiaa29*, in addition to a higher rate of shrunken and higher chalkiness seed under high-temperature conditions (*t*-test, *P* < 0.01) (Table 1). A similar phenotype was found in *OsIAA29*-RNAi plants (Additional file 1: Fig. S2 and Table S4). Observation of the random seeds (3-21 DAP) of *osiaa29* showed that during the first 5 DAF, there is no obvious difference between ZH11 and *osiaa29* seeds. After 7 DAF, the *osiaa29* seeds became shrunken which persists until 21 DAF, whereas the seeds of ZH11 are filled under high temperature. Conversely, the phenotypes of *osiaa29* lines were same as ZH11 under normal conditions (Fig. 2D, E). Histological analysis revealed that abnormal embryonic development in *osiaa29*. The specific performance is embryo developmental abnormalities swelled or no embryo formation compared with ZH11 control. In addition, whatever big embryo or no embryo, their endosperm both performance loose distribution, indicating that *OsIAA29* influences the development of endosperm and embryo in rice (Fig. 2F). For mature seeds, we found the *osiaa29* exhibited a higher rate of chalkiness and shrunken grain under natural high temperature conditions. Conversely, the chalkiness of OE29 lines was same as ZH11 and the shrunken grain rate of OE29 was declined compared with ZH11 (Fig. 2G and Table 1). To further investigate functions of *OsIAA29*, we used scanning electron microscope (SEM) to examine the seed of *osiaa29* lines. Compared with ZH11, the starch grains of *osiaa29* seeds with severe phenotype were smaller and rounder (Fig. 2H). These suggest that the main reasons for the decrease of grain yield were the increased rate of shrunken seed and the decrease of seed weight.

**Table 1:** Agronomic traits of *OsIAA29* mutants under normal and high temperature

Lines	Normal temperature			High temperature		
	Shrunken grain rate (%)	1000-grain weight (g)	Chalkiness rate (%)	Shrunken grain rate (%)	1000-grain weight (g)	Chalkiness rate (%)
ZH11	10.89 ± 1.94	24.99 ± 0.11	10.17 ± 2.66	24.84 ± 12.66	24.93 ± 0.21	24.50 ± 3.34
*osiaa29-1*	25.07 ± 4.62*	22.93 ± 0.19**	30.50 ± 3.56**	93.27 ± 5.22**	21.00 ± 0.20**	84.00 ± 0.41**
*osiaa29-2*	30.57 ± 4.01**	23.01 ± 0.08**	33.33 ± 1.93**	98.19 ± 1.30**	22.16 ± 0.26**	73.50 ± 2.50**
OE29-1	23.44 ± 3.32**	23.61 ± 0.15**	31.50 ± 2.00**	5.86 ± 3.30**	24.09 ± 0.42	25.66 ± 1.93

Data are presented as means standard error (SE) of three biological replicates. *P*-values were calculated using two-tailed *t*-test

*P < 0.05, **P < 0.01

### Seed Storage Substances were Altered in *osiaa29* Seed

By analyzing the storage substance of *OsIAA29* transgenic lines, we found significant reduction in the content of total starch and glutelin in *osiaa29* than WT under high temperature. Whereas, the content of glutelin and prolamin in *osiaa29* lines was the same as ZH11 under the normal conditions, the content of starch was decreased (Table 2). At high temperature, the decrease of starch content was more obvious. This suggested that *OsIAA29* might be involve in the accumulation of seed storage substances by regulating the synthesis of total starch. Moreover, higher percentage of shrunken seed and lower 1000-grain weight of *osiaa29* in the high temperature might be probably caused by the insufficient filling of starch and glutelin. In conclusion, *OsIAA29* might be involved in the grain filling stage of rice, especially at high temperatures.

**Table 2:** Protein and starch contents of *osiaa29* seed under normal and high temperature

Lines	Normal temperature			High temperature		
	Content of starch (%)	Content of glutelin (%)	Content of prolamin (%)	Content of starch (%)	Content of glutelin (%)	Content of prolamin (%)
ZH11	83.12 ± 0.90	8.06 ± 0.20	1.66 ± 0.03	82.85 ± 0.46	7.97 ± 0.02	1.65 ± 0.01
*osiaa29-1*	72.88 ± 2.01**	8.18 ± 0.08	1.68 ± 0.07	68.09 ± 0.75**	6.47 ± 0.68*	1.73 ± 0.19
*osiaa29-2*	75.45 ± 0.74**	8.28 ± 0.07	1.79 ± 0.01	67.97 ± 0.73**	6.40 ± 0.72*	1.74 ± 0.12
OE29-1	82.24 ± 0.79	6.60 ± 0.15**	1.43 ± 0.26	71.00 ± 2.20**	9.30 ± 0.27**	1.17 ± 0.17

Data are presented as means standard error (SE) of three biological replicates. *P*-values were calculated using two-tailed *t*-test

*P < 0.05, **P < 0.01

### OsIAA29 Interacts with Auxin Response Factors OsARF17

**Fig. 3:**
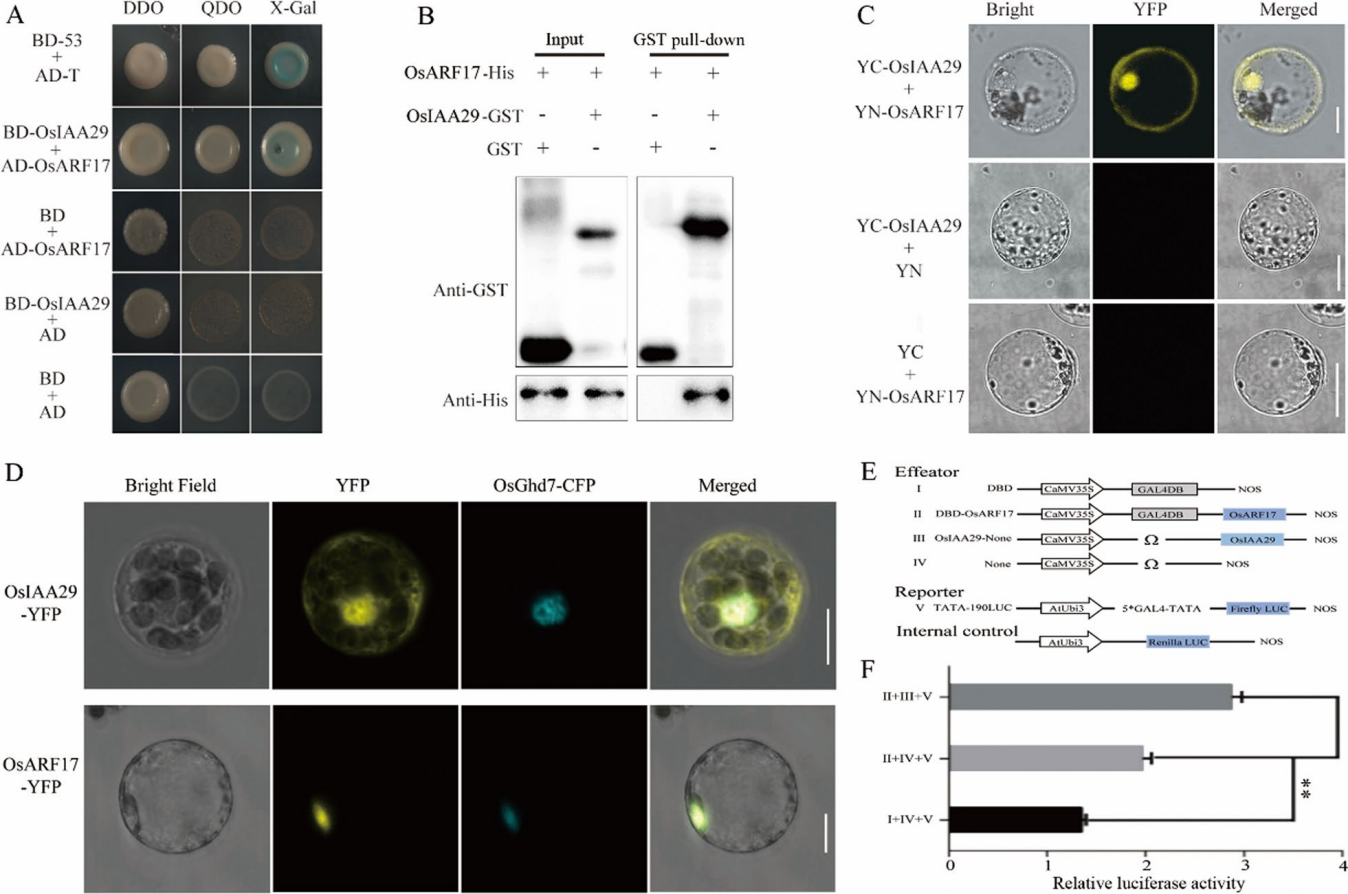
Interaction between OsIAA29 and OsARF17. **A** Yeast two-hybrid assay. The full-length OsIAA29 cDNA was cloned into a vector bearing the DNA binding domain (BD), and the full-length cDNA of OsARF17 was cloned into a vector bearing an activation domain (AD). The transformants were grown on DDO (SD/-Leu/-Trp), QDO (SD/-Leu/-Trp/-His/-Ade), and QDO with X-α-Gal plates. **B** Pull-down assays showing that there was a direct interaction between GST-OsIAA29 and His-OsARF17 in vitro. The recombinant proteins were expressed in the *Escherichia coli* BL21 strain (DE3) and Glutathione beads were used for pull-down. GST-fused free protein was used as the control. Western blotting was performed using anti-GST or anti-His antibody. (Sigma-Aldrich). **C** BiFC assays of OsIAA29 and OsARF17. OsIAA29-cYFP and OsARF17-nYFP interacted to form a functional CFP in rice protoplast cells. Scale bars are 10 μm. **D** Subcellular localization of OsIAA29 and OsARF17. OsGhd7, the nuclear marker. Bar = 5 μm. **E**–**f** Transient expression analysis of 35S-5xGAL4-TATA-LUC activity in rice protoplasts. **E** The vector of luciferase combination. **F** Dual luciferase analysis of the activation activity of OsARF17. OsARF17 or OsARF17/OsIAA29 was co-transfected with 35S-5xGAL4-TATA-LUC. 35S-5xGAL4-TATA-LUC used as control. The LUC/REN ratio was shown to indicate the expression level of the 35S-5xGAL4-TATA-LUC. The values in each column are the mean (± SD) of three replicates. Significant differences were determined using Student’s *t*-test (* *P* < 0.05; ** *P* < 0.01)

As it has been reported that AUX/IAA can interact with ARF, we then investigated whether any OsARFs protein can interact with OsIAA29. It was found that OsARF17 may interact with OsIAA29 based on Y2H (Fig. 3A and Additional file 1: Fig. S3). We next performed an in vitro GST Pull-Down assay to examine the interaction between OsIAA29 and OsARF17. OsIAA29 was fused to a GST tag, while OsARF17 was fused to a His tag. The results of GST pull-down assay further confirmed that OsIAA29 could interacted with OsARF17 in vitro (Fig. 3B).

To further confirm the direct interaction of OsIAA29 with OsARF17, BiFC analysis was carried out in rice protoplasts. Yellow fluorescence generated from the interaction between OsIAA29-YFP^N^ and OsARF17-YFP^C^ was detected, confirming the formation of a heterodimer by OsIAA29 and OsARF17 in the nucleus and cytoplasm. Co-localization of OsIAA29 and OsARF17 and their overlapping signals that occurred predominantly in the nucleus indicated that they could form a heterodimer in the nucleus (Fig. 3C).

We then transiently expressed OsIAA29 or OsARF17 protein with the fusion of yellow fluorescent protein (YFP) in protoplast to investigate the subcellular localization pattern of the two genes. 35S::Ghd7-CFP was used as a nuclear marker (Xue et al. 2008). The fluorescent signals generated by OsIAA29-YFP were detected in the nucleus and cytoplasm in the protoplast, while the fluorescent signals generated by OsARF17-YFP, were also detected in the nucleus in the protoplast (Fig. 3D). The results indicated that OsIAA29 and OsARF17 proteins were indeed localized in the nucleus.

After determining the localization pattern of OsIAA29 and OsARF17, we guess whether the OsIAA29 can regulate auxin signaling by interacting with OsARF17, we co-expressed 35S::Gal4DBD-OsARF17 and 35S::OsIAA29 construct in protoplast cells isolated from rice leaf sheath. We found that transcriptional activation activity of OsARF17 was enhanced by OsIAA29 (Fig. 3E, F), suggesting that OsIAA29 might regulate auxin signal pathways by enhancing OsARF17.

### OsIAA29-OsARF17 Affects Key Factors in Substance Accumulation

**Fig. 4:**
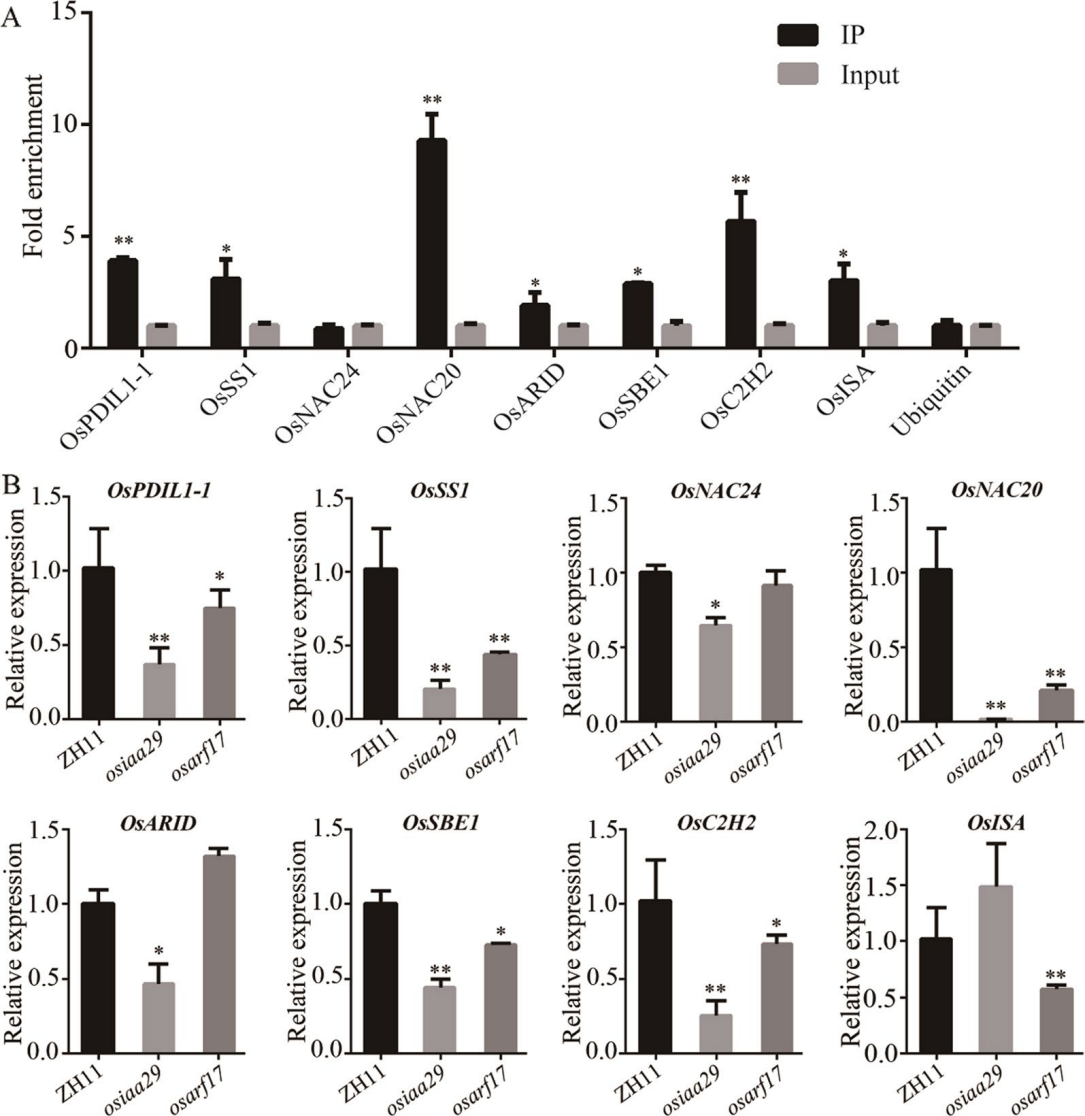
Identification of OsARF17 direct target genes in rice. **A** ChIP-PCR verification of OsARF17-bound regions. The data are the mean values (± SD) of fold-enrichment from *n* = 3 technical replicates. **B** qRT-PCR analysis of expression levels of the target genes in *osiaa29* and *osarf17* compared with ZH11. Ubiquitin was used as the reference gene. Data are presented as means standard error (SE) of three biological replicates. *P*-values were calculated using two-tailed *t*-test. *P < 0.05, **P < 0.01

Owing to a significantly reduction in the content of total starch and glutelin in *osiaa29*, we used qRT-PCR to analyze the expression of 39 reported genes associated with starch synthesis and protein synthesis in *osiaa29* mutants. The results showed that there were 19 genes upregulated and 11 genes downregulated in *osiaa29* compare with WT (Additional file 1: Table S5). The previous studies revealed that ARF bind motifs including ‘TGTCTC’, ‘TGTCGG’, and ‘TGTCNN’, which implied that OsARF17 probably binds to the promoters of its downstream targets through the TGTC-box motif (Zemlyanskaya et al. 2016). There were 21 genes containing ARG bind motifs among the 30 differentially expressed genes using PLACE (A Database of Plant Cis-acting Regulatory DNA Elements). To study the functional mechanisms of OsIAA29 and OsARF17, we performed ChIP assays using the anti-FLAG antibody with 7-DAP seeds of OE17 (the overexpression lines of *OsARF17*, Additional file 1: Fig. S4B-C). The expression of the OsARF17-FLAG fusion proteins was investigated by western blot analysis to validate the effectiveness of the FLAG tags. The result identified that seven genes associated with starch synthesis, protein synthesis, and transcription factor about synthesis from 21 preferably bound genes as the potential target genes of OsIAA29 and OsARF17 (Fig. 4A). The results of qRT-PCR assay demonstrated that the expression of these genes (*OsPDIL1-1*, *OsSS1*, *OsNAC20*, *OsSBE1*, and *OsC2H2*) were down-regulated in *osiaa29* and *osarf17* (OsARF17 CRISPR transgenic lines, Additional file 1: Fig. S4A) (Fig. 4B). EMSA assays demonstrated that OsARF17-GST bounds to the promoter of *OsPDIL1-1* and regulated the expression of these genes (Additional file 1: Fig. S5).

### OsIAA29 Regulates Auxin Signal Pathway Under High Temperature

**Fig. 5:**
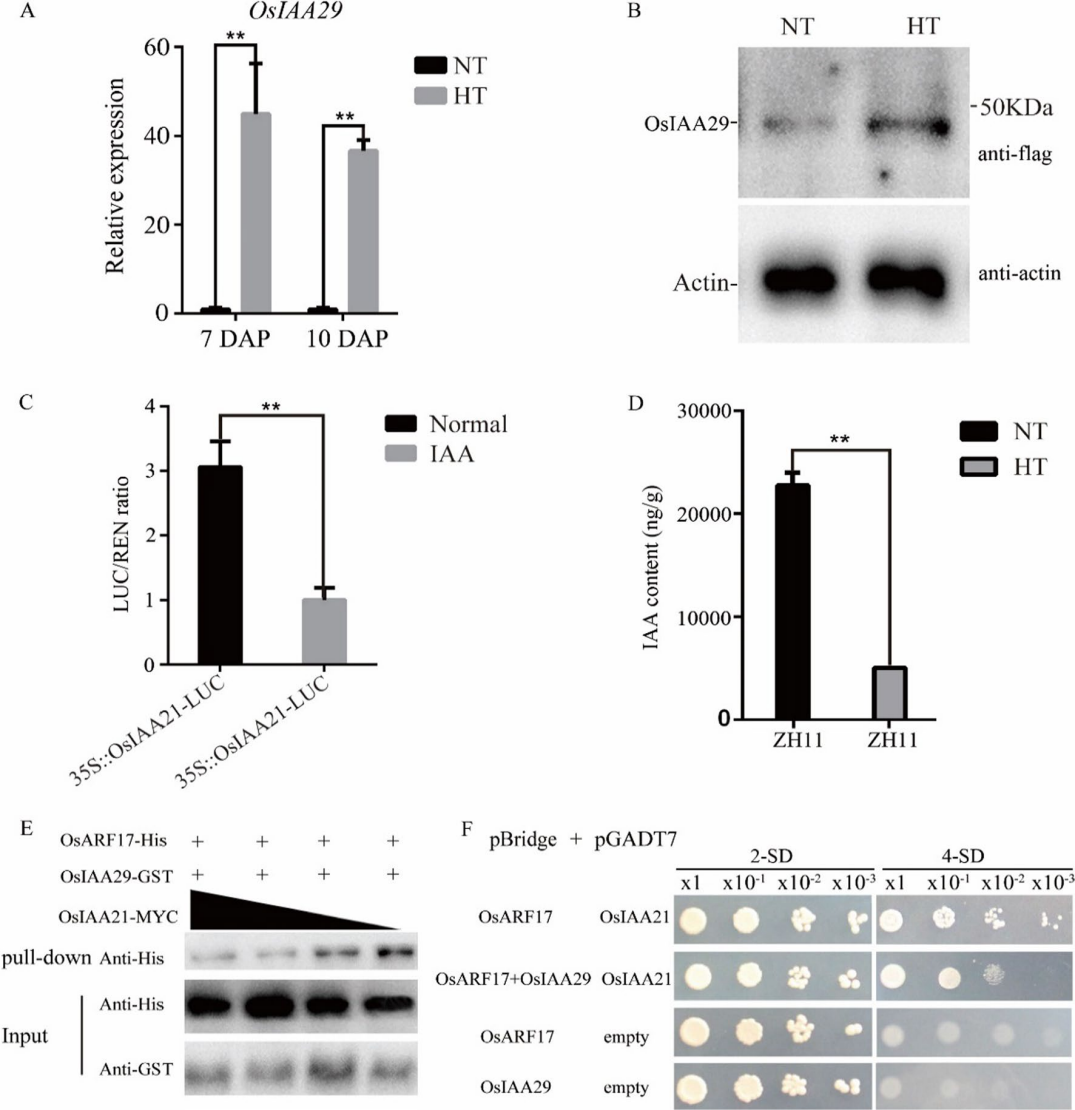
OsIAA29 competes with OsIAA21 in the binding to OsARF17. **A** The expression of *OsIAA29* in developing seeds of ZH11 under high temperature and normal temperature. **B** Western-Blot detects the GFP tagged OsIAA29 in OE29; vector is *pZmUbi*::*OsIAA29-GFP*. **C** Dual luciferase analysis of the effect of auxin to OsIAA21. **D** MS analysis of changes in auxin content in rice seeds under high temperature/normal temperature conditions. **E** Immunoblot of pull-down/protein competition assay OsARF17-His and OsIAA29-GST with increasing amounts of OsIAA21-MYC. The anti‐His antibody is indicated pull-down at the top panel, while the input shown at the bottom panel. **F** Yeast three-hybrid assay analyzing the OsIAA21-OsARF17 interaction in the presence or absence of co-expressed OsIAA29. Co-transformants were spotted on 2-SD (-Leu-Trp) medium to check for viability and on 4-SD (-Met-His-Leu-Trp) medium to test the interaction and competition. Data are mean standard error (SE) for three replicates. *P < 0.05, **P < 0.01. *P*-values produced by two-tailed Student’s *t*-test

As *osiaa29* has a more severe phenotype under high temperature than that under normal conditions (Fig. 2), we seek to investigate whether OsIAA29 was involved in heat stress response. We isolated 7 DAP rice seeds of ZH11 and OE lines under high temperature and normal conditions for qRT-PCR and western blot analysis. The qRT-PCR results showed that *OsIAA29* was higher expressed in rice seeds (7 DAP and 10 DAP) under high temperature compared with natural conditions (Fig. 5A). Western blot results further suggested that the accumulation of OsIAA29 protein under high temperatures (Fig. 5B). These results suggest that *OsIAA29* could be induced by heat stress.

In rice, OsARF17 not only interacts with OsIAA29, but also interacts with OsIAA21 (Shen et al. 2010). OsIAA21 is canonical AUX/IAA protein (Additional file 1: Fig. S6) and the level of OsIAA21 protein was decreased by IAA using Dual-luc (Fig. 5C). Meanwhile, the IAA content was obviously reduced in the seed of ZH11 under high temperature using LC–MS-MS (Fig. 5D), indicating that OsIAA21 could be accumulated in high temperature. Therefore, we examined whether OsIAA29 could compete with OsIAA21 to interact with OsARF17 at high temperatures. In vitro pull-down assays indicated that increasing the level of OsIAA21-MYC clearly reduced the interaction OsIAA29-GST between with OsARF17-His, suggesting that OsIAA29 could compete with OsIAA21 in the interaction with OsARF17 (Fig. 5E). This result was also confirmed by yeast three-hybrid assays (Fig. 5F). These results show that the OsIAA29 regulates the auxin signal pathway in high temperatures by competing with OsIAA21 to OsARF17.

## Discussion

### OsIAA29 is a Special Function Non-canonical AUX/IAAs Protein

AUX/IAA regulates the function of ARF to mediate the auxin signaling pathway (Salehin et al. 2015). Previous researches in *Arabidopsis* and rice have suggested a key role for IAA in organogenesis, apical dominance, organogenesis, embryo formation, morphogenesis, vascular differentiation, and light and gravity perception (Tian and Reed 1999; Fukaki et al. 2006). The AUX/IAA proteins have four highly conserved subdomains (domains I‐IV), the functions of every subdomain have been reported by previous studies. Thus, AUX/IAA may have functional redundancy because similar domains in a protein impart similar functions. For example, In *Arabidopsis*, IAA3, IAA14, IAA19, IAA28, and IAA15 directly activates the transcription of LBD16 and LBD29 to induce lateral root formation (Kim et al. 2020). In addition, there are some non-canonical AUX/IAA proteins (six in *Arabidopsis*, five in rice), which have no typical domain I or II. It has been recently reported that these non‐canonical AUX/IAA proteins could also regulate auxin signaling (Lv et al. 2020). Treatment of non- canonical AUX/IAA proteins seedlings with auxin promoted an accumulation of proteins over time (Cao et al. 2019). In this study, we found though OsIAA29 has no typical subdomain I and II (Fig. 1C). Meanwhile, OsIAA29 form a heterodimer with OsARF17 and are predominantly expressed at the early and middle stage of rice seed development. OsIAA29, a non-canonical AUX/IAA protein, may regulate auxin signaling in the seed development of rice.

### OsIAA29 May be a Competitor Mediating Heat Stress Response During Grain Filling

**Fig. 6:**
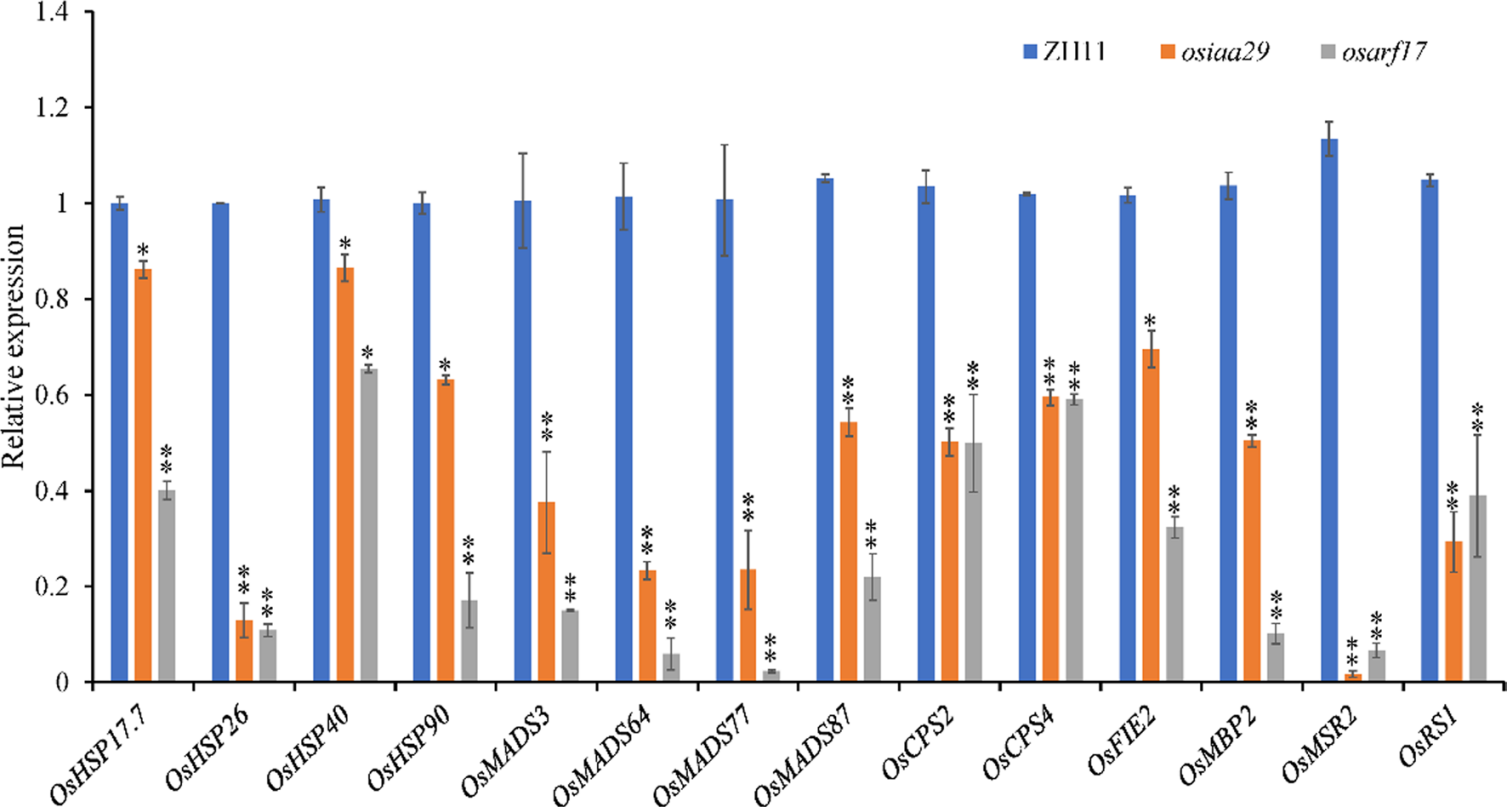
Quantitative detection of high temperature stress response genes in rice. Expression levels of related genes in 7 DAP seeds by qRT-PCR analysis. Values are presented as means standard error (SE) (n = 3). *P*-values were calculated using two-tailed *t*-test. *P < 0.05, **P < 0.01. Primers are listed in Additional file 1: Table S1

The phytohormone auxin plays an important role in heat stress-induced thermomorphogenesis, including stem (hypocotyl) elongation and leaf hyponasty (Kim et al. 2020). Auxin transport and auxin signal transduction was also altered in response to high temperature (Huai et al. 2018). In high temperatures, the auxin contents were significantly reduced in seeds, the expression of *OsIAA29* was significantly higher than normal conditions in ZH11. The overexpression lines also have more higher protein accumulation in heat stress (Fig. 5), demonstrating that higher proportion of incompletely filled grains than ZH11 in high temperature. The findings indicate that OsIAA29 responds to heat stress. A previous study showed that OsARF17 could interact with OsIAA21 and this result was confirmed (Kieffer et al. 2010). Further research finds OsIAA29 and OsIAA21 compete with OsARF17. The auxin signing pathway was reduced due to reduction of auxin content, but OsIAA29 induced by heat stress, could activate this pathway to guarantee seed normal development by competing with OsIAA21 binding OsARF17 (Fig. 5). QRT-PCR results show that the expression level of a series of heat response genes including *OsHSPs*, *OsMADSs*, *OsCPS*, and *OsMSR2*, were significantly down-regulated in the transgenic lines of *osiaa29* and *osarf17* (Fig. 6). A previous study has shown that HSPs as chaperones, play a pivotal role in conferring biotic and abiotic stress tolerance. which can maintain plant protein functional conformation and prevent non-native proteins from aggregation (Gu et al. 2019). Os*MADSs* encoded transcription factors are essential for the regulation of various aspects of flower development which are regulated under moderate and severe heat stress (Chen et al. 2016; Guo et al. 2021). OsCPS is the biosynthesis of Gibberellic acid gene while the GA signaling pathway is involved in the regulation of cell elongation and morphogenesis under heat stress (Tanaka et al. 2006). OsMSR2, a calmodulin-like protein, is strongly induced by drought, cold and heat stress, which can bind and sense Ca^2+^ in plant cells (Xu et al. 2011). We hereby speculate that OsIAA29 not only was strongly induced by heat stress, but also transfer the heat stress signaling to other heat stress gene by the OsIAA29-OsARF17 module.

### The OsIAA29-OsARF17 Module Regulates the Biosynthesis of Storage Substances in Rice Seed

OsIAA29 accelerates the accumulation of starch and protein by promoting IAA signal transduction and participates in the filling of inferior spikelet under moderate soil drying (Teng et al. 2022). A series of experiments in our study suggested that OsIAA29 interacts with OsARF17 in vitro and vivo. The 1000-grain weight of *osarf17* was reduced and the shrunken seed rate increased obviously, similar to that in *osiaa29* lines (Additional file 1: Fig. S4 and Table S5). OsARF17 was a typical transcription factor which can directly bind “AuxREs” motif to activate the expression of downstream genes, suggesting the possibility that OsIAA29 and OsARF17 to regulate seeds development. Sucrose crosses the extracellular space to endosperm by SWEETs and SUTs, then is converted into starch granule by starch synthases and other starch-related enzymes (Yang et al. 2018). Among them, a study shows that SUTs is the direct target of RhARF7 in rose (Liang et al. 2020), the promoter of OsSWEEATs and OsSUTs have “AuxREs” motif or potential “AuxREs” motif. So, we assume that OsIAA29 and OsARF17 may influences sucrose transporters to regulate the accumulation of material.

Furthermore, ChIP and qRT-PCR analysis showed significant enrichment of starch biosynthesis. The expression levels of several genes related to starch synthesis and seed storage proteins were reduced in the *osiaa29* and *osarf17*. This includes (*NAC020*, *NAC024*, *OsISA*, *OsSS1*, *OsSBE1*, and *OsPDIL1-1*), the mutant of *NAC020* lines have no detrimental effect but *NAC020*/*NAC026* both double mutants had significantly reduced protein and starch content cause floury endosperm and decreased kernel length, kernel width, and kernel thickness (Wang et al. 2020a). *NAC024*, encoding a NAC family transcription factor, which acts on OsMED15a to regulate grain size and grain weight (Dwivedi et al. 2019). OsPDIL1-1 is a protein desulphated isomerase-like enzyme that is homologous with human PDI, *ospdil1-1* causes floury and white-core endosperm and endoplasmic reticulum stress response (Han et al. 2012; Xia et al. 2018). OsARF17 could directly bind to the promoter of these genes (Fig. 4C). These suggest that OsIAA29-OsARF17 modulates the process of storage-substance accumulation by regulating the expression of multiple genes associated with starch biosynthesis.

Based on the above results, we propose the possible model (Additional file 1: Fig. S7). Under normal condition, both OsIAA29 and OsIAA21 could interact with OsARF17, but auxin promotes the stability of OsIAA29 proteins and the degradation of Aux/IAA proteins, with OsIAA29 mainly binding to OsARF17, the activity of OsARF17 was released to the regulation of seed development. The rice seeds development and maturation are normal. When heat stress was introduced, the auxin content was significantly decreased, the expression of OsIAA29 was induced by heat stress. Additionally, due to low level auxin content, auxin induces the degradation of canonical AUX/IAA proteins through the 26S proteasome-dependent ubiquitin was paused, leading to the accumulation of the OsIAA21 protein. OsIAA29 is like a “rescuer”, OsIAA29 and OsIAA21 compete with OsARF17, which balances the activity of OsARF17 between activation and inhibition, maintaining rice seeds development and maturation (Additional file 1: Fig. S7). In conclusion, OsIAA29 and auxin may regulate the transcription of OsARF17 required for seed development and grain filling.

## Materials and Methods

### Plant Material and Growth Conditions

For generation of *OsIAA29* and *OsARF17* mutants, the specific single-guide RNA (sgRNA) was designed using CRISPR-P (http://crispr.hzau.edu.cn/CRISPR/) and was cloned into a binary vector pYLCRISPR/Cas9-MH (Ma et al. 2015). The RNAi construct with 350 bp specific nucleotides from the *OsIAA29* coding sequence were amplified using cDNA of ZH11. Then, the sequences were introduced into the target vector pDS1301 as previously reported (Wang et al. 2015). To generate the over-expression plants, the cDNA fragments of *OsIAA29* and *OsARF17* without a stop codon were amplified, and the eGFP or 3 × Flag coding sequence was fused at the 3’ end of cDNA. The fused sequences were cloned into the binary vector pCAMBIA1301U (driven by maize ubiquitin promoter), respectively. To construct the promoter vector of *OsIAA29* and *OsARF17*, a 2000-bp fragment of the 5′ upstream region of *OsIAA29* and a 2000-bp fragment of the 5′ upstream region of *OsARF17* were cloned into the binary vector pDX2181 (Wang et al. 2020b). The recombinant constructs were transferred into calli of the cultivar ‘zhonghua11’ by *Agrobacterium tumefaciens*-mediated transformation using the strain EHA105 (Wu et al. 2003).

The transgenic lines were cultivated in paddy fields of Huazhong Agricultural University under natural rice growing seasons during May to October 2019. The experiment of high-temperature stress was conducted following the method reported in our previous study (Ren et al. 2021). A temperature of 35 °C was set as the heat damage temperature of rice, and the heat damage accumulated temperature per hour (TH_i_) was calculated as $$TH_{i} = \left\{ {\begin{array}{*{20}l} {T_{I} - 35} \hfill & {T_{I} \ge 35} \hfill \\ 0 \hfill & {T_{I} < 35} \hfill \\ \end{array} } \right.$$ (T_i_ is the ambient temperature at i hour); heat damage hours during filling stage (HS) was calculated as $$H_{s} = \mathop \sum \limits_{i = 0}^{n} H_{i} \left( {H_{i} = \left\{ {\begin{array}{*{20}l} 1 \hfill & {T_{i} \ge 35} \hfill \\ 0 \hfill & {T_{i} < 35} \hfill \\ \end{array} } \right.} \right)$$⁠; and heat damage accumulated temperature during filling stage (TS) was calculated as ${T}_{s}={\sum }_{i=0}^{n}{TH}_{i}$⁠. The Ts during 0–7 DAP (TS7) and Hs during 0–7 DAP (HS7) was also calculated. In order to perform the high temperature stress in paddy field, we sowed four batches of rice and each batch was spaced half a month apart according to the meteorological data. The temperature data of the growing area during rice reproductive stages are shown in Additional file 1: Fig. S1. We finally selected two batches of rice according to the flowering time and the temperature data. Since two batches of plants (including *osiaa29*, *OsIAA29*-RNAi, *OsIAA29*-OE, and WT) were blooming on large scale on 3 July 2019 and 5 August 2019, respectively, we marked most of the flowering spikelets and the immature seeds used in the experiment were all from the marked spikelet. We calculated the heat accumulation temperature from the 3 July 2019 and 5 August 2019, respectively.

### Yeast Assay

For yeast one‐hybrid, coding sequence of *OsARF17* was amplified using special primers and was then linked to pGADT7. A genomic fragment upstream of the start codon of *OsIAA29* was amplified and subcloned into the pHIS2 vector. These constructs were co-transformed into the yeast strain Y187. The interaction was screened using synthetic dropout (-Leu/-Trp/-His) medium containing 3-AT.

For yeast two-hybrid, the coding sequences of *OsIAA29*, *OsIAA21*, and *OsARF17* were cloned into pGBKT7 (BD) and pGADT7 (AD), respectively. The recombinational AD and BD plasmids were co-transformed into yeast strain AH109 and transformants were selected on SD (-Leu/Trp) medium. The colonies were tested onto SD (‐Trp‐Leu‐His‐Ade) containing X‐α‐gal. Yeast transformants with BD53 and AD-T were used as the positive control, and colonies co-transformed with BD and AD empty vector was selected as the negative control (Xiong et al. 2019).

The yeast three‐hybrid experiment was performed according to the methods in a previously study (Licitra and Liu 1996). The complete CDS of *OsIAA29* and *OsIAA21* were amplified (Additional file 1: Table S1) and fused with pBridge-ARF17 plasmid to generate pBridge‐OsARF17‐OsIAA29 and pBridge‐OsARF17‐OsIAA21, respectively. The yeast strain Y2H Gold was transformed with a pair of plasmids, pBridge‐OsARF17‐OsIAA21 and OsIAA29‐pGADT7, pBridge‐OsARF17‐OsIAA29 and OsIAA21‐pGADT7. Protein interactions were confirmed using selective SD (‐Met‐His‐Leu‐Trp) medium.

### BiFC and Subcellular Localization Analysis

For the BiFC assay, the CDS of *OsIAA29* and *OsARF17* was cloned into pVYNE and pVYCE, respectively (Waadt et al. 2008). For the subcellular localization assay, the CDS of *OsIAA29* and *OsARF17* was cloned into the pM999-35S vector. The 35S::Ghd7-CFP was used as a nuclear localization marker (Xue et al. 2008). Then, the plasmids were co-transformed into rice protoplasts according to the method (Shen et al. 2017; Liu et al. 2022). The confocal laser scanning microscope (TCS SP8, Leica, Wetzlar, Germany) was used to detect fluorescent signals.

### In Vitro Pull-Down Assay

The CDS of *OsIAA29*, *OsIAA21*, and *OsARF17* was cloned into pGEX4T-GST, pGEX4T-MYC and pET28a-His, respectively. The fusion plasmids pGEX4T-OsIAA29-GST, pGEX4T-OsIAA21-MYC, and pET28a-OsARF17-His were expressed in the *Escherichia coli* BL21 strain (DE3). The pull-down assay was performed according to the previous method (Xiong et al. 2019). The recombinant protein and Glutathione Beads were simultaneously incubated overnight at 4 °C. GST‐fused free protein was used as the control. The beads, washed ten times with GST pull-down buffer, were boiled in protein loading buffer at 99 °C for 10 min. Western blot was performed using anti-GST or anti-His antibody (Sigma-Aldrich).

### Chromatin Immunoprecipitation (ChIP)

ChIP was performed as described (Stojanova et al. 2015). Briefly, seeds at 7 DAP were harvested and immediately cross-linked in 1% formaldehyde under vacuum for 30 min, and 2 g of tissues for each sample was used for chromatin isolation, which were then subject to six rounds of sonication (Bioruptor, Diagenode) 6 min each (30 s on/off). The DNA was immunoprecipitated by anti-FLAG® M2 magnetic beads (Sigma, M8823). To detect the specific DNA targets, the precipitated DNA and input DNA were applied for qPCR analysis. The enrichment value was normalized to that of the input sample. The significance of differences was estimated using Student’s *t*-test.

### Transient transcription Dual-Luciferase (LUC) Assays

For the effectors, the CDS of *OsIAA29*, *OsIAA21*, and *OsARF17* was cloned into yeast GAL4 binding domain vectors (GAL4BD) or “None”. The 35S-GAL4-Fluc were used as reporters, and AtUbi::rLUC was used as an internal control. The relative luciferase activity was calculated as the ratio between fLUC and rLUC according to method (Ren et al. 2021).

### RNA Isolation and qRT-PCR Analysis

Immature seeds were collected and ground in liquid nitrogen. Total RNA was isolated using TRIzol reagent (Invitrogen, California, USA). First-strand cDNA was reverse transcribed using HiScript II Reverse Transcriptase (Vazyme, Nanjing, China). Quantitative reverse transcription PCR (qRT-PCR) was performed on an QuantStudio 7 Flex Real-Time PCR System (Applied Biosystems, Carlsbad, CA, USA). Ubiquitin (Ubq) was used as an internal reference. Each experiment was performed with three biological replicates. The relative expression level of genes were detected using gene-specific primers (Additional file 1: Table S1) and estimated via the 2^−ΔΔCT^ method (Livak and Schmittgen 2001). *OsACTIN1* (LOC_Os03g50885) was used as an internal control and each experiment was executed using three biological replicates.

### Detection of ß-Glucuronidase (GUS) Activity

Expression profiling were detected using various organs of p*OsIAA29*::GUS transgenic lines as previously methods (Wang et al. 2020b). Twenty-five 7-day seeds and 10-day seeds from five transgenic plants were collected and directly immersed in GUS staining solution. The organs, incubated 12–16 h at 37 °C, incubated 12–16 h at 37 °C, were cleared for in 70% ethanol and photographed using an Olympus SZX16 Zoom Stereo Microscope (Olympus, Tokyo, Japan, http://www.olympus-global.com/en/) stocked with the camera (Olympus E-330).

### Microscopy Analysis

The brown rice seeds of WT, *osiaa29*, OE29, and the Ri29 plants coverslips were fixed with 2% PFA/ 2.5% glutaralydehyde, which were then cut transversely with a knife and coated with gold under vacuum conditions. Samples were observed using an SEM (JSM-6390LV). Scanning electron microscope (SEM) was performed as described previously (Zhou et al. 2017).

### Electrophoretic Mobility Shift Assay (EMSA)

For EMSA, according to the previous methods (Shen et al. 2020). Briefly, the forward single-stranded DNA oligonucleotides were mixed with equimolar amount of the complementary strand. Proteins were incubated with probe for 30 min at 25 degrees in the final solution containing. The obtained products were then resolved in 6% (v/v) native acrylamide gels using 0.5 × TBE buffer under an electric field of 120 V for 1 h.

### Supplementary Information

**Additional file 1: Figure S1**. Ambient temperature of growing areas during rice grain filling stage. 35 °C is marked as yellow dotted lines and 40 °C is marked as red dotted lines; TS, heat damage accumulated temperature during the whole filling stage; HS, heat damage hours during the whole filling stage; T_S7_, heat damage accumulated temperature during 0–7 DAP; H_S7_, heat damage hours during 0-7 DAP. **Figure S2**. Agronomic traits of *OsIAA29*-RNAi (Ri29-1, Ri29-2) under high temperature. (A) Relative expression level of *OsIAA29*-RNAi lines in ZH11. (B–C) 1000 grain weight and shrunken seed rate of WT and Ri-29 lines. (D) The WT and Ri-29 lines images of grains of mature seeds. Bar = 1cm. **Figure S3**. The results of yeast two-hybrid analysis of the interaction between *OsIAA29* and *OsARFs*. The full-length OsIAA29 cDNA was cloned into a vector bearing the DNA binding domain (BD), and the full-length cDNA of OsARFs were cloned into a vector bearing an activation domain (AD). The transformants were grown on DDO (SD/-Leu/-Trp) and QDO (SD/-Leu/-Trp/-His/-Ade) plates. **Figure S4**. Identification of OsARF17 CRISPR line (*osarf17*) and overexpressed plant (OE17). (A) Mutation sites in *osarf17-1* and *osarf17-2*, as compared with wild-type (WT) sequences, protospacer-adjacent motif sequences are shown in bold, and inserted or deleted nucleotides are indicated in red. (B) Relative expression level of overexpression materials lines in ZH11. (C) Detection of FLAG fusion protein in ZH11 and overexpression lines. Total proteins extracted from developing caryopses at 7-DAP were used for western blot analysis with an anti-FLAG antibody. (D–E) 1000 grain weight and shrunken seed rate of WT and *osarf17* lines. Data are presented as means standard error (SE) of five biological replicates. *P*-values were calculated using two-tailed t-test. *P < 0.05, **P < 0.01. Figure S5. EMSA analysis OsARF17 bindings to the promoter of the target gene (OsPDIL1-1). **Figure S6**. OsIAA21 was a canonical AUX/IAA protein. (A) The structure of OsIAA21. The blue boxes represent the exons, the line represents the intron and the white boxes the UTRs. (B) Schematic diagram of the domains of OsIAA21. (C) Compared with other canonical AUX/IAA. **Figure S7**. Schematic diagram of the OsIAA29 response heat stress in rice endosperm. OsIAA29 plays upstream regulatory roles in endosperm development, and the accumulation of storage substances. When the seeds under heat stress conditions, the auxin level is low, the OsIAA29 protein is induced by heat stress, which leads to a high level of OsIAA29 protein, and OsARF17 are compete by OsIAA21 and OsIAA29 protein. When the seeds under normal conditions, the auxin level is normal, both OsIAA29 and OsIAA21 are degraded by a 26S proteasome-dependent mechanism, but OsIAA29 protein is slowly degraded, and the transcription activity of OsARF17 was released. **Table S1**. Primers for functional analysis of *OsIAA29*. **Table S2**. Detection of potential off-target sites for the sgRNAs. Table S3 Agronomic traits of *osiaa29* under normal and high temperature in 2018. **Table S4** Agronomic traits of WT and *OsIAA29*-RNAi lines under high temperature in 2019. Table S5 Agronomic traits of *osarf17* under normal and high temperature in 2021. **Table S6**. qRT-PCR analysis of selected genes in *osiaa29-1*.

## Data Availability

All datasets generated for this study are included in the article/ Additional  file 1.

## Abbreviations

DAP: Day after pollination
GUS: β-Glucuronidase
CDS: Coding sequence
GFP: Green fluorescent protein
YFP: Yellow fluorescent protein
SEM: Scanning electron microscope
Y2H: Yeast two-hybrid
sgRNA: Single-guide RNA
ChIP: Chromatin immunoprecipitation

## References

[CR1] Ai H, Bellstaedt J, Bartusch KS, Eschen-Lippold L, Babben S, Balcke GU, Tissier A, Hause B, Andersen TG, Delker C, Quint M (2023). Auxin-dependent regulation of cell division rates governs root thermomorphogenesis. EMBO J.

[CR2] Basunia MA, Nonhebel HM, Backhouse D, McMillan M (2021). Localised expression of OsIAA29 suggests a key role for auxin in regulating development of the dorsal aleurone of early rice grains. Planta.

[CR3] Cao M, Chen R, Li P, Yu Y, Zheng R, Ge D, Zheng W, Wang X, Gu Y, Gelova Z, Friml J, Zhang H, Liu R, He J, Xu T (2019). TMK1-mediated auxin signalling regulates differential growth of the apical hook. Nature.

[CR4] Chen C, Begcy K, Liu K, Folsom JJ, Wang Z, Zhang C, Walia H (2016). Heat stress yields a unique MADS box transcription factor in determining seed size and thermal sensitivity. Plant Physiol.

[CR5] Chen JY, Zhang HW, Zhang HL, Ying JZ, Ma LY, Zhuang JY (2018). Natural variation at *qHd1* affects heading date acceleration at high temperatures with pleiotropism for yield traits in rice. BMC Plant Biol.

[CR6] Dhatt BK, Paul P, Sandhu J, Hussain W, Irvin L, Zhu F, Adviento-Borbe MA, Lorence A, Staswick P, Yu H, Morota G, Walia H (2021). Allelic variation in rice *Fertilization Independent Endosperm 1* contributes to grain width under high night temperature stress. New Phytol.

[CR7] Dwivedi N, Maji S, Waseem M, Thakur P, Kumar V, Parida SK, Thakur JK (2019). The mediator subunit OsMED15a is a transcriptional co-regulator of seed size/weight-modulating genes in rice. Biochimica Et Biophysica Acta Gene Regul Mech.

[CR8] Fukaki H, Taniguchi N, Tasaka M (2006). PICKLE is required for SOLITARY-ROOT/IAA14-mediated repression of ARF7 and ARF19 activity during *Arabidopsis* lateral root initiation. Plant J.

[CR9] Gu LL, Li MZ, Wang GR, Liu XD (2019). Multigenerational heat acclimation increases thermal tolerance and expression levels of Hsp70 and Hsp90 in the rice leaf folder larvae. J Therm Biol.

[CR10] Gu X, Si F, Feng Z, Li S, Liang D, Yang P, Yang C, Yan B, Tang J, Yang Y, Li T, Li L, Zhou J, Li J, Feng L, Liu JY, Yang Y, Deng Y, Wu XN, Zhao Z, Wan J, Cao X, Song X, He Z, Liu J (2023). The OsSGS3-tasiRNA-OsARF3 module orchestrates abiotic-biotic stress response trade-off in rice. Nat Commun.

[CR11] Guo M, Zhang X, Liu J, Hou L, Liu H, Zhao X (2020). OsProDH negatively regulates thermotolerance in rice by modulating proline metabolism and reactive oxygen species scavenging. Rice (n y).

[CR12] Guo F, Huang Y, Qi P, Lian G, Hu X, Han N, Wang J, Zhu M, Qian Q, Bian H (2021). Functional analysis of auxin receptor OsTIR1/OsAFB family members in rice grain yield, tillering, plant height, root system, germination, and auxinic herbicide resistance. New Phytol.

[CR13] Hakata M, Wada H, Masumoto-Kubo C, Tanaka R, Sato H, Morita S (2017). Development of a new heat tolerance assay system for rice spikelet sterility. Plant Methods.

[CR14] Han X, Wang Y, Liu X, Jiang L, Ren Y, Liu F, Peng C, Li J, Jin X, Wu F, Wang J, Guo X, Zhang X, Cheng Z, Wan J (2012). The failure to express a protein disulphide isomerase-like protein results in a floury endosperm and an endoplasmic reticulum stress response in rice. J Exp Bot.

[CR15] Huai J, Zhang X, Li J, Ma T, Zha P, Jing Y, Lin R (2018). SEUSS and PIF4 coordinately regulate light and temperature signaling pathways to control plant growth. Mol Plant.

[CR16] Kieffer M, Neve J, Kepinski S (2010). Defining auxin response contexts in plant development. Curr Opin Plant Biol.

[CR17] Kim SH, Bahk S, An J, Hussain S, Nguyen NT, Do HL, Kim JY, Hong JC, Chung WS (2020). A gain-of-function mutant of IAA15 inhibits lateral root development by transcriptional repression of LBD genes in *Arabidopsis*. Front Plant Sci.

[CR18] Liang Y, Jiang C, Liu Y, Gao Y, Lu J, Aiwaili P, Fei Z, Jiang CZ, Hong B, Ma C, Gao J (2020). Auxin regulates sucrose transport to repress petal abscission in rose (*Rosa hybrida*). Plant Cell.

[CR19] Licitra EJ, Liu JO (1996). A three-hybrid system for detecting small ligand-protein receptor interactions. PNAS.

[CR20] Liu K, Wang X, Liu H, Wu J, Liang F, Li S, Zhang J, Peng X (2022). OsAT1, an anion transporter, negatively regulates grain size and yield in rice. Physiol Plantarum.

[CR21] Livak KJ, Schmittgen TD (2001). Analysis of relative gene expression data using real-time quantitative PCR and the 2(-Delta Delta C(T)) method. Methods.

[CR22] Lv B, Yu Q, Liu J, Wen X, Yan Z, Hu K, Li H, Kong X, Li C, Tian H, De Smet I, Zhang XS, Ding Z (2020). Non-canonical AUX/IAA protein IAA33 competes with canonical AUX/IAA repressor IAA5 to negatively regulate auxin signaling. EMBO J.

[CR23] Ma X, Zhang Q, Zhu Q, Liu W, Chen Y, Qiu R, Wang B, Yang Z, Li H, Lin Y, Xie Y, Shen R, Chen S, Wang Z, Chen Y, Guo J, Chen L, Zhao X, Dong Z, Liu YG (2015). A robust CRISPR/Cas9 system for convenient, high-efficiency multiplex genome editing in monocot and dicot plants. Mol Plant.

[CR24] Ma M, Shen SY, Bai C, Wang WQ, Feng XH, Ying JZ, Song XJ (2023). Control of grain size in rice by TGW3 phosphorylation of OsIAA10 through potentiation of OsIAA10-OsARF4-mediated auxin signaling. Cell Rep.

[CR25] Ma F, Zhang F, Zhu Y, Lan D, Yan P, Wang Y, Hu Z, Zhang X, Hu J, Niu F, Liu M, He S, Cui J, Yuan X, Yan Y, Wu S, Cao L, Bian H, Yang J, Li Z, Luo X (2023a) Auxin signaling module OsSK41-OsIAA10-OsARF regulates grain yield traits in rice. J Integr Plant Biol 10.1111/jipb.13484 36939166

[CR26] Nevame AYM, Emon RM, Malek MA, Hasan MM, Alam MA, Muharam FM, Aslani F, Rafii MY, Ismail MR (2018). Relationship between high temperature and formation of chalkiness and their effects on quality of rice. Biomed Res Int.

[CR27] Nie DM, Ouyang YD, Wang X, Zhou W, Hu CG, Yao J (2013). Genome-wide analysis of endosperm-specific genes in rice. Gene.

[CR28] Peng S, Huang J, Sheehy JE, Laza RC, Visperas RM, Zhong X, Centeno GS, Khush GS, Cassman KG (2004). Rice yields decline with higher night temperature from global warming. PNAS.

[CR29] Ramos Baez R, Nemhauser JL (2021). Expansion and innovation in auxin signaling: where do we grow from here?. Development.

[CR30] Ren Y, Huang Z, Jiang H, Wang Z, Wu F, Xiong Y, Yao J (2021). A heat stress responsive NAC transcription factor heterodimer plays key roles in rice grain filling. J Exp Bot.

[CR31] Salehin M, Bagchi R, Estelle M (2015). SCFTIR1/AFB-based auxin perception: mechanism and role in plant growth and development. Plant Cell.

[CR32] Shen C, Wang S, Bai Y, Wu Y, Zhang S, Chen M, Guilfoyle TJ, Wu P, Qi Y (2010). Functional analysis of the structural domain of ARF proteins in rice (*Oryza sativa* L.). J Exp Bot.

[CR33] Shen J, Liu J, Xie K, Xing F, Xiong F, Xiao J, Li X, Xiong L (2017). Translational repression by a miniature inverted-repeat transposable element in the 3' untranslated region. Nat Commun.

[CR34] Shen C, Liu H, Guan Z, Yan J, Zheng T, Yan W, Wu C, Zhang Q, Yin P, Xing Y (2020). Structural insight into DNA recognition by CCT/NF-YB/YC complexes in plant photoperiodic flowering. Plant Cell.

[CR35] Stojanova ZP, Kwan T, Segil N (2015). Epigenetic regulation of *Atoh1* guides hair cell development in the mammalian cochlea. Development.

[CR36] Tanaka N, Matsuoka M, Kitano H, Asano T, Kaku H, Komatsu S (2006). *gid1*, a gibberellin-insensitive dwarf mutant, shows altered regulation of probenazole-inducible protein (PBZ1) in response to cold stress and pathogen attack. Plant Cell Environ.

[CR37] Teng Z, Yu H, Wang G, Meng S, Liu B, Yi Y, Chen Y, Zheng Q, Liu L, Yang J, Duan M, Zhang J, Ye N (2022). Synergistic interaction between ABA and IAA due to moderate soil drying promotes grain filling of inferior spikelets in rice. Plant J.

[CR38] Tian Q, Reed JW (1999). Control of auxin-regulated root development by the *Arabidopsis thaliana* SHY2/IAA3 gene. Development.

[CR39] Waadt R, Schmidt LK, Lohse M, Hashimoto K, Bock R, Kudla J (2008). Multicolor bimolecular fluorescence complementation reveals simultaneous formation of alternative CBL/CIPK complexes in planta. Plant J.

[CR40] Wang X, Zhou W, Lu Z, Ouyang Y, Yao J (2015). A lipid transfer protein, OsLTPL36, is essential for seed development and seed quality in rice. Plant Sci.

[CR41] Wang J, Chen Z, Zhang Q, Meng S, Wei C (2020). The NAC transcription factors OsNAC20 and OsNAC26 regulate starch and storage protein synthesis. Plant Physiol.

[CR42] Wang X, Yan X, Tian X, Zhang Z, Wu W, Shang J, Ouyang J, Yao W, Li S (2020). Glycine- and proline-rich protein OsGPRP3 regulates grain size and quality in rice. J Agric Food Chem.

[CR43] Wu C, Li X, Yuan W, Chen G, Kilian A, Li J, Xu C, Li X, Zhou DX, Wang S, Zhang Q (2003). Development of enhancer trap lines for functional analysis of the rice genome. Plant J.

[CR44] Xia K, Zeng X, Jiao Z, Li M, Xu W, Nong Q, Mo H, Cheng T, Zhang M (2018). Formation of protein disulfide bonds catalyzed by OsPDIL1;1 is mediated by microRNA5144-3p in rice. Plant Cell Physiol.

[CR45] Xiong Y, Ren Y, Li W, Wu F, Yang W, Huang X, Yao J (2019). NF-YC12 is a key multi-functional regulator of accumulation of seed storage substances in rice. J Exp Bot.

[CR46] Xu GY, Rocha PS, Wang ML, Xu ML, Cui YC, Li LY, Zhu YX, Xia X (2011). A novel rice calmodulin-like gene, *OsMSR2*, enhances drought and salt tolerance and increases ABA sensitivity in *Arabidopsis*. Planta.

[CR47] Xu J, Henry A, Sreenivasulu N (2020). Rice yield formation under high day and night temperatures—a prerequisite to ensure future food security. Plant Cell Environ.

[CR48] Xue W, Xing Y, Weng X, Zhao Y, Tang W, Wang L, Zhou H, Yu S, Xu C, Li X, Zhang Q (2008). Natural variation in *Ghd7* is an important regulator of heading date and yield potential in rice. Nat Genet.

[CR49] Yang J, Luo D, Yang B, Frommer WB, Eom JS (2018). SWEET11 and 15 as key players in seed filling in rice. New Phytol.

[CR50] Zemlyanskaya EV, Wiebe DS, Omelyanchuk NA, Levitsky VG, Mironova VV (2016). Meta-analysis of transcriptome data identified TGTCNN motif variants associated with the response to plant hormone auxin in *Arabidopsis thaliana* L. J Bioinf Comput Biol.

[CR51] Zhou W, Wang X, Zhou D, Ouyang Y, Yao J (2017). Overexpression of the 16-kDa alpha-amylase/trypsin inhibitor RAG2 improves grain yield and quality of rice. Plant Biotechnol J.

