# Supplementary material for: Heat Stress Responsive Aux/IAA Protein, OsIAA29 Regulates Grain Filling Through OsARF17 Mediated Auxin Signaling Pathway
Source: Rice (N Y). 2024 Feb 19;17:16. doi: 10.1186/s12284-024-00694-z (PMC10876508; doi:10.1186/s12284-024-00694-z)
Supplement: Supplementary file 1 — Additional file 1: Figure S1. Ambient temperature of growing areas during rice grain filling stage. 35 °C is marked as yellow dotted lines and 40 °C is marked as red dotted lines; TS, heat damage accumulated temperature during the whole filling stage; HS, heat damage hours during the whole filling stage; TS7, heat damage accumulated temperature during 0–7 DAP; HS7, heat damage hours during 0-7 DAP. Figure S2. Agronomic traits of OsIAA29-RNAi (Ri29-1, Ri29-2) under high temperature. (A) Relative expression level of OsIAA29-RNAi lines in ZH11. (B–C) 1000 grain weight and shrunken seed rate of WT and Ri-29 lines. (D) The WT and Ri-29 lines images of grains of mature seeds. Bar = 1cm. Figure S3. The results of yeast two-hybrid analysis of the interaction between OsIAA29 and OsARFs. The full-length OsIAA29 cDNA was cloned into a vector bearing the DNA binding domain (BD), and the full-length cDNA of OsARFs were cloned into a vector bearing an activation domain (AD). The transformants were grown on DDO (SD/-Leu/-Trp) and QDO (SD/-Leu/-Trp/-His/-Ade) plates. Figure S4. Identification of OsARF17 CRISPR line (osarf17) and overexpressed plant (OE17). (A) Mutation sites in osarf17-1 and osarf17-2, as compared with wild-type (WT) sequences, protospacer-adjacent motif sequences are shown in bold, and inserted or deleted nucleotides are indicated in red. (B) Relative expression level of overexpression materials lines in ZH11. (C) Detection of FLAG fusion protein in ZH11 and overexpression lines. Total proteins extracted from developing caryopses at 7-DAP were used for western blot analysis with an anti-FLAG antibody. (D–E) 1000 grain weight and shrunken seed rate of WT and osarf17 lines. Data are presented as means standard error (SE) of five biological replicates. P-values were calculated using two-tailed t-test. *P < 0.05, **P < 0.01. Figure S5. EMSA analysis OsARF17 bindings to the promoter of the target gene (OsPDIL1-1). Figure S6. OsIAA21 was a canonical AUX/IAA protein. (A) [file 12284_2024_694_MOESM1_ESM.doc]

**Supplementary materials**

**H****eat stress responsive Aux/IAA protein, OsIAA29 regulates rice grain filling through OsARF17 mediated auxin signaling pathway**

Zhanghao Chen ^1#^, Wei Zhou ^3#^, Xianyu Guo ^1^, Sheng Ling, Wang Li, Xin Wang ^2*^, Jialing Yao ^1*^

1 College of Life Science and Technology, Huazhong Agricultural University, Wuhan 430070, China

2 Key Laboratory of Molecular Biology and Genetic Engineering of Jiangxi Province, School of Life Sciences, Nanchang University, Nanchang 330031, China

3 College of Life Sciences, Xinyang Normal University, Xinyang 464000, China

First authors: Zhanghao Chen and Wei Zhou

* Corresponding authors: Xin Wang, Jialing Yao

E-mail: wangxin@ncu.edu.cn; yaojlmy@mail.hzau.edu.cn

**Supplementary figures and tables**


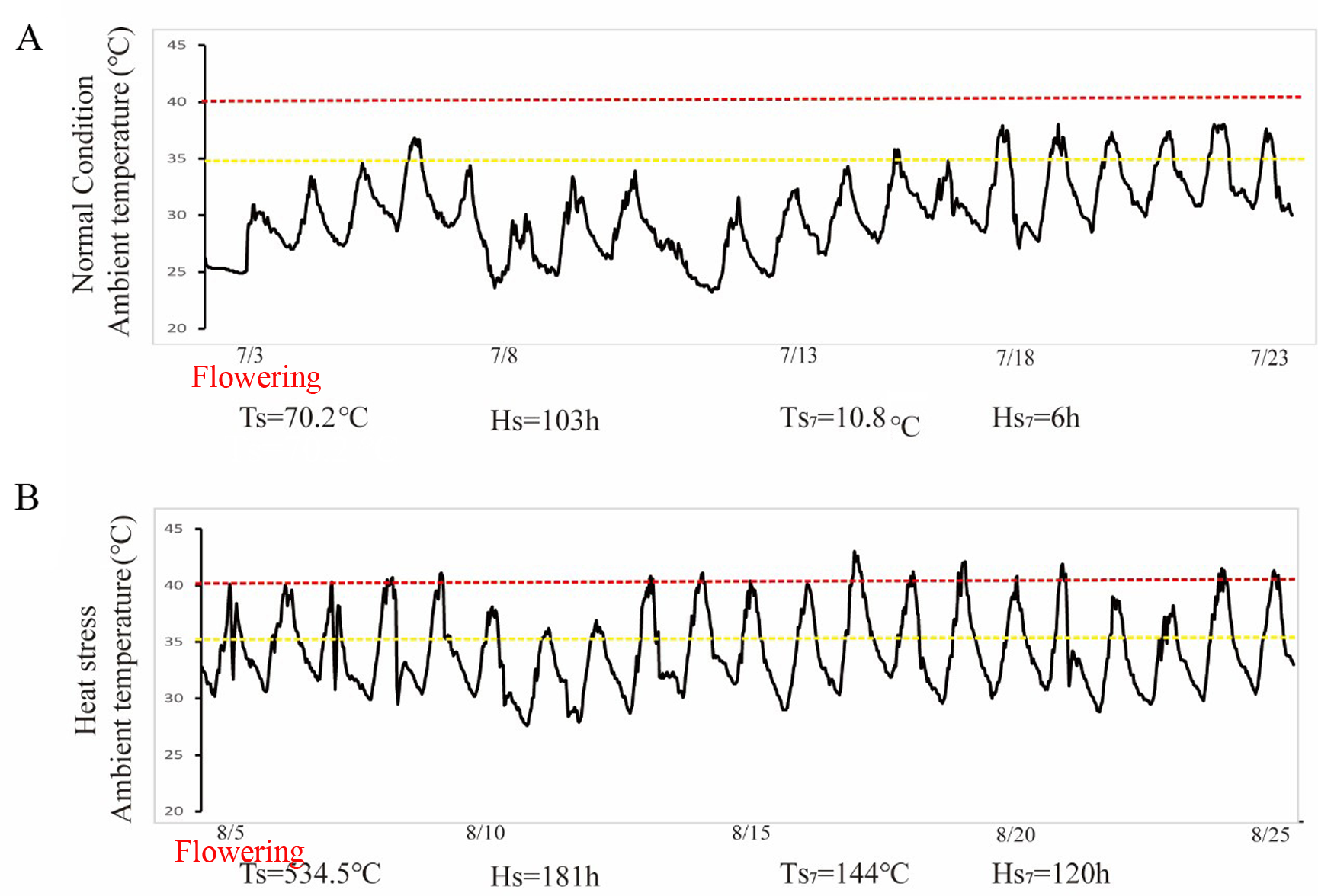


**Figure S1. Ambient temperature of growing areas during rice grain filling stage**. The rice flowered around 3 July 2019 and 5 August 2019, respectively. 35℃ is marked as yellow dotted lines and 40℃ is marked as red dotted lines; T_S_, heat damage accumulated temperature during the whole filling stage; H_S_, heat damage hours during the whole filling stage; T_S7_, heat damage accumulated temperature during 0-7 DAP; H_S7_, heat damage hours during 0-7 DAP.


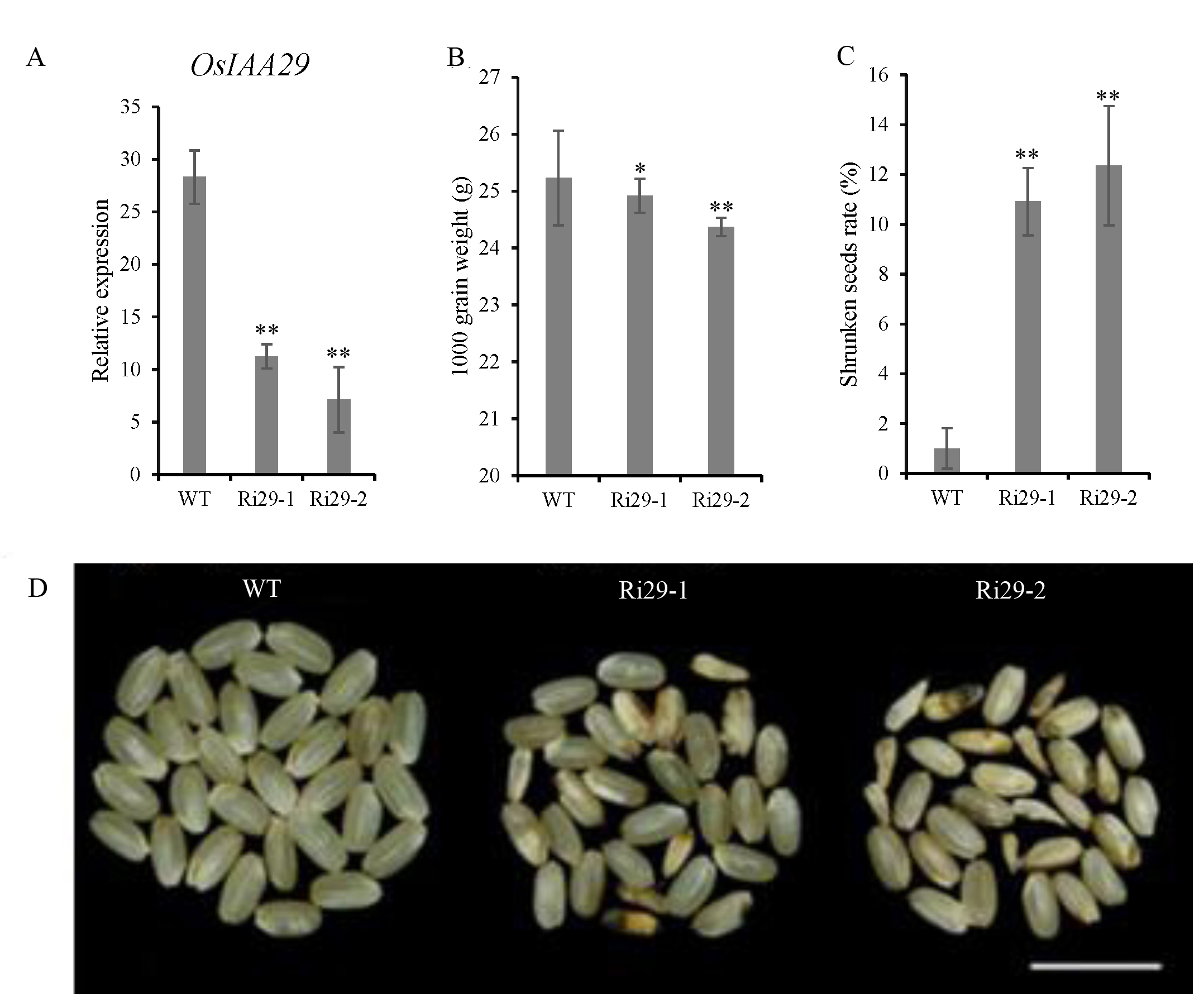


**Figure S2. Agronomic traits of *OsIAA29*-RNAi (Ri29-1, Ri29-2) under high temperature.** (A) Relative expression level of *OsIAA29*-RNAi lines in ZH11. (B-C) 1000 grain weight and shrunken seed rate of WT and Ri-29 lines. (D) The WT and Ri-29 lines images of grains of mature seeds. Bar=1cm. Data are presented as means standard error (SE) of three biological replicates. *P*-values were calculated using two-tailed *t*-test. **P* < 0.05, ***P* < 0.01.


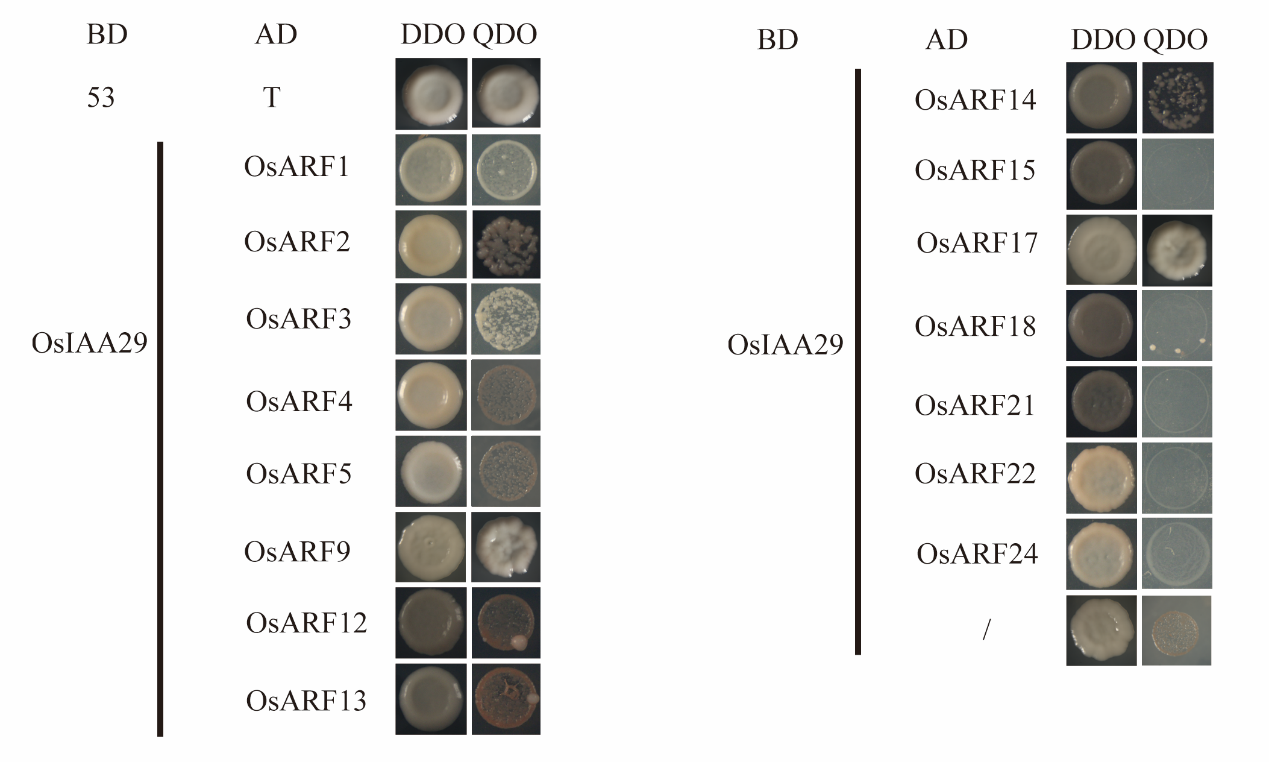


**Figure S3 The results of yeast two-hybrid analysis of the interaction between OsIAA29 and OsARFs.** The full-length OsIAA29 cDNA was cloned into a vector bearing the DNA binding domain (BD), and the full-length cDNA of OsARFs were cloned into a vector bearing an activation domain (AD). The transformants were grown on DDO (SD/-Leu/-Trp) and QDO (SD/-Leu/-Trp/-His/-Ade) plates.


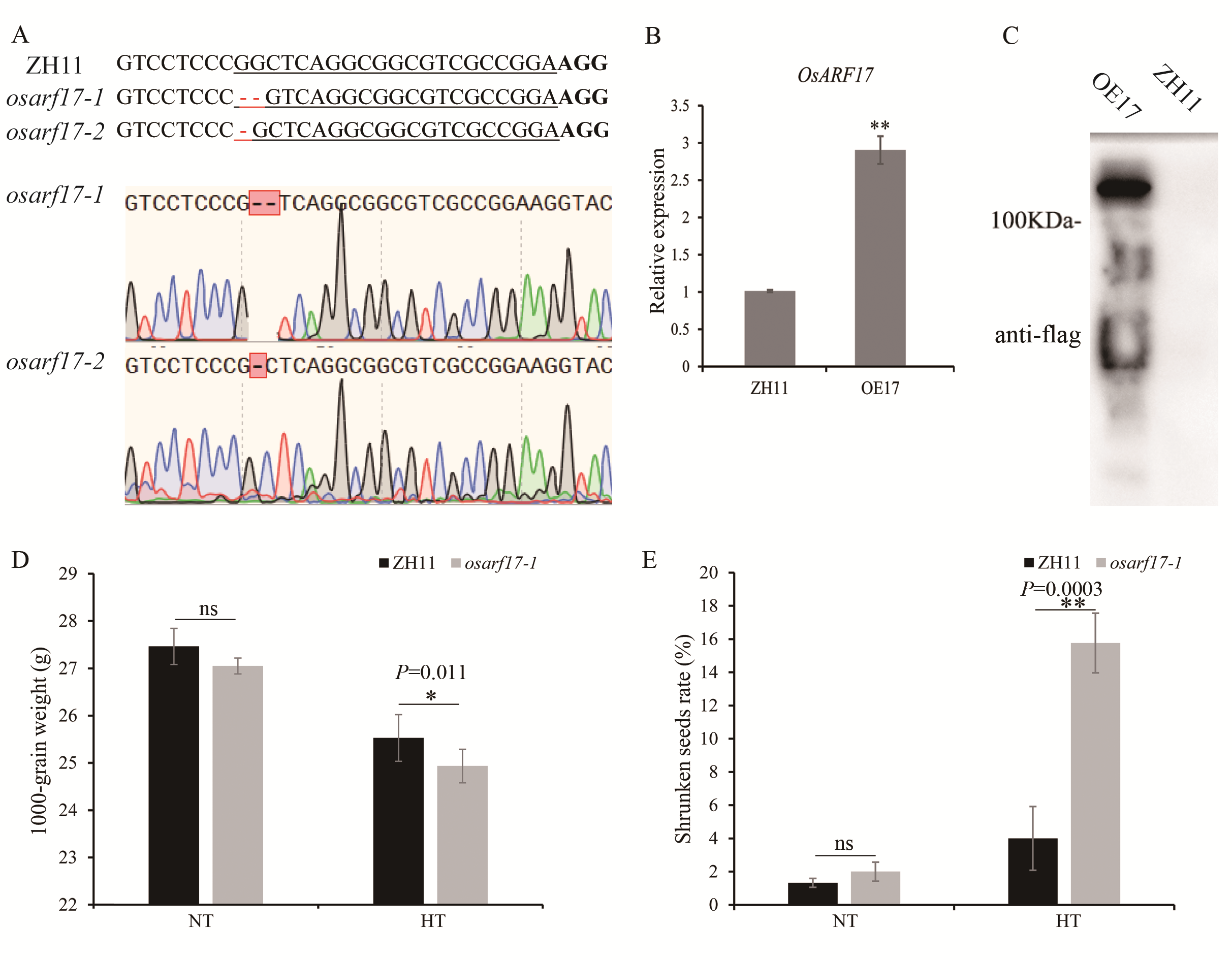


**Figure S4. Identification of OsARF17 CRISPR lines (*osarf17*) and overexpressed plant (OE17).** (A) Mutation sites in *osarf17*-1 and *osarf17*-2, as compared with wild-type (WT) sequences, protospacer-adjacent motif sequences are shown in bold, and inserted or deleted nucleotides are indicated in red. (B) Relative expression level of overexpression materials lines in ZH11. (C) Detection of FLAG fusion proteins in ZH11 and overexpression lines. Total proteins extracted from developing caryopses at 7-DAP were used for western blot analysis with an anti-FLAG antibody. (D-E) 1000 grain weight and shrunken seed rate of WT and *osarf17* lines. Data are presented as means standard error (SE) of five biological replicates. *P*-values were calculated using two-tailed *t*-test. **P* < 0.05, ***P* < 0.01.


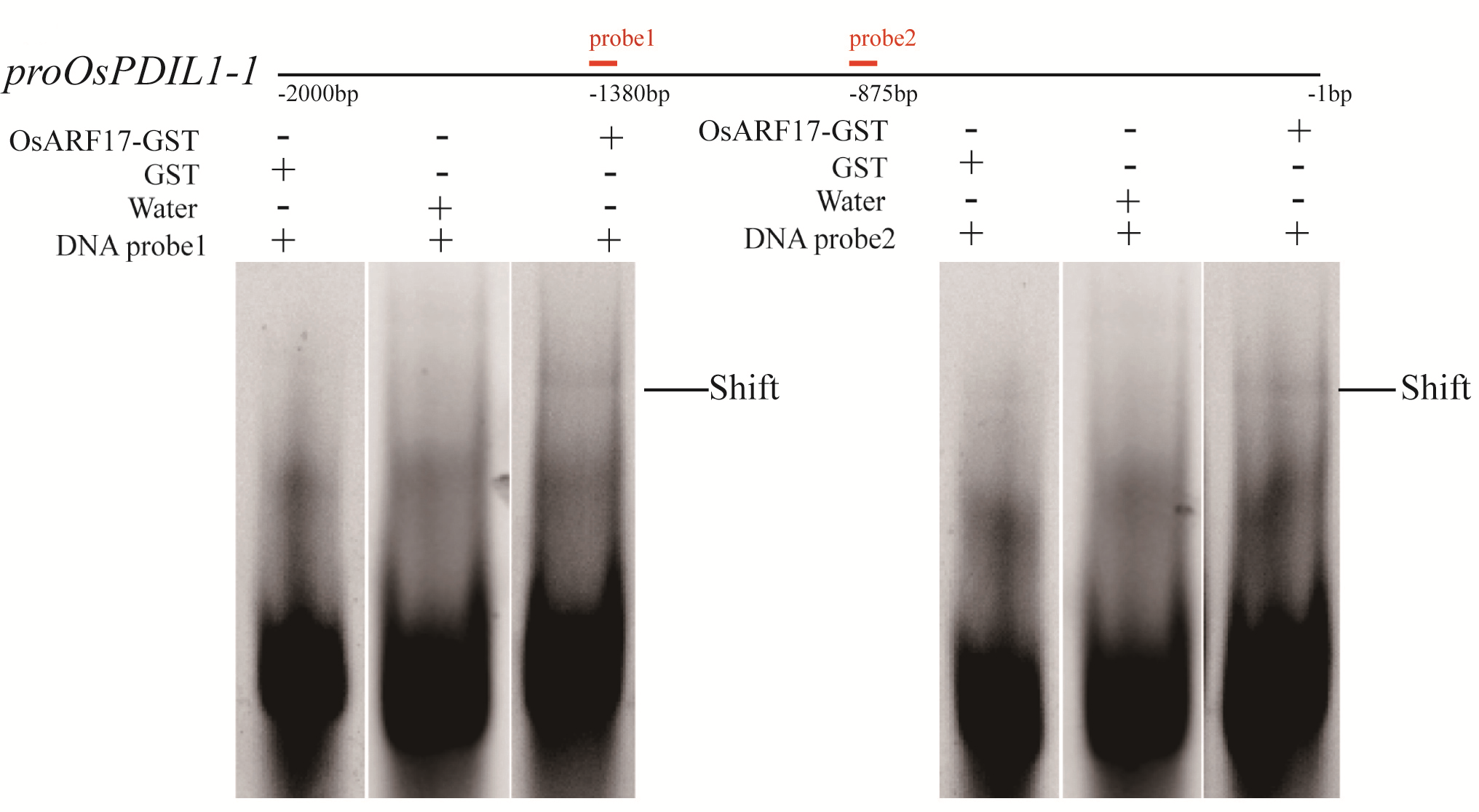


**Figure S5. EMSA analysis OsARF17 bindings to the promote of the target gene (*OsPDIL1-1*).**


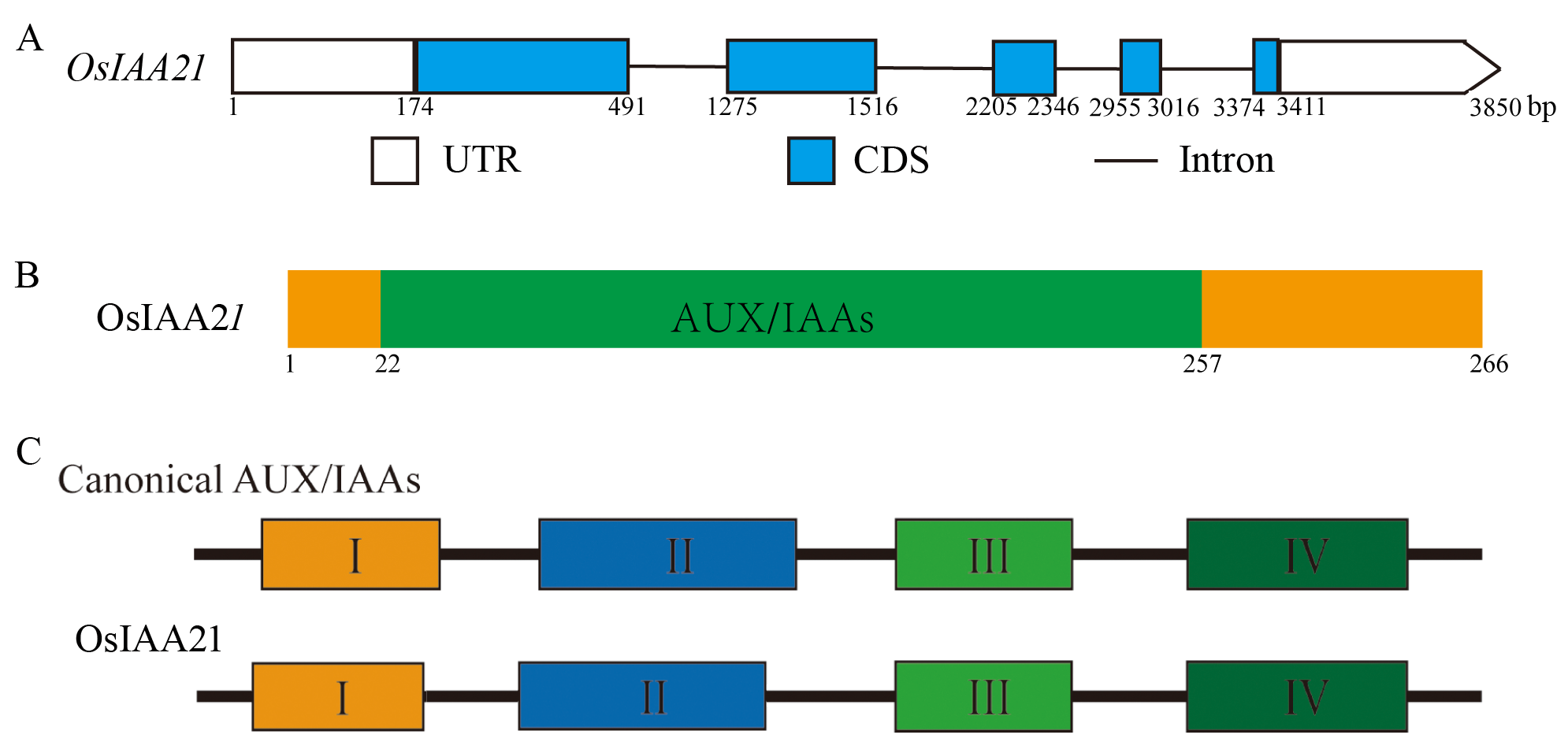
**Figure S6. OsIAA21 was a canonical AUX/IAA protein.** (A) The structure of OsIAA21. The blue boxes represent the exons, the line represents the intron and the white boxes the UTRs. (B) Schematic diagram of the domains of OsIAA21. (C) Compared with other canonical AUX/IAA.


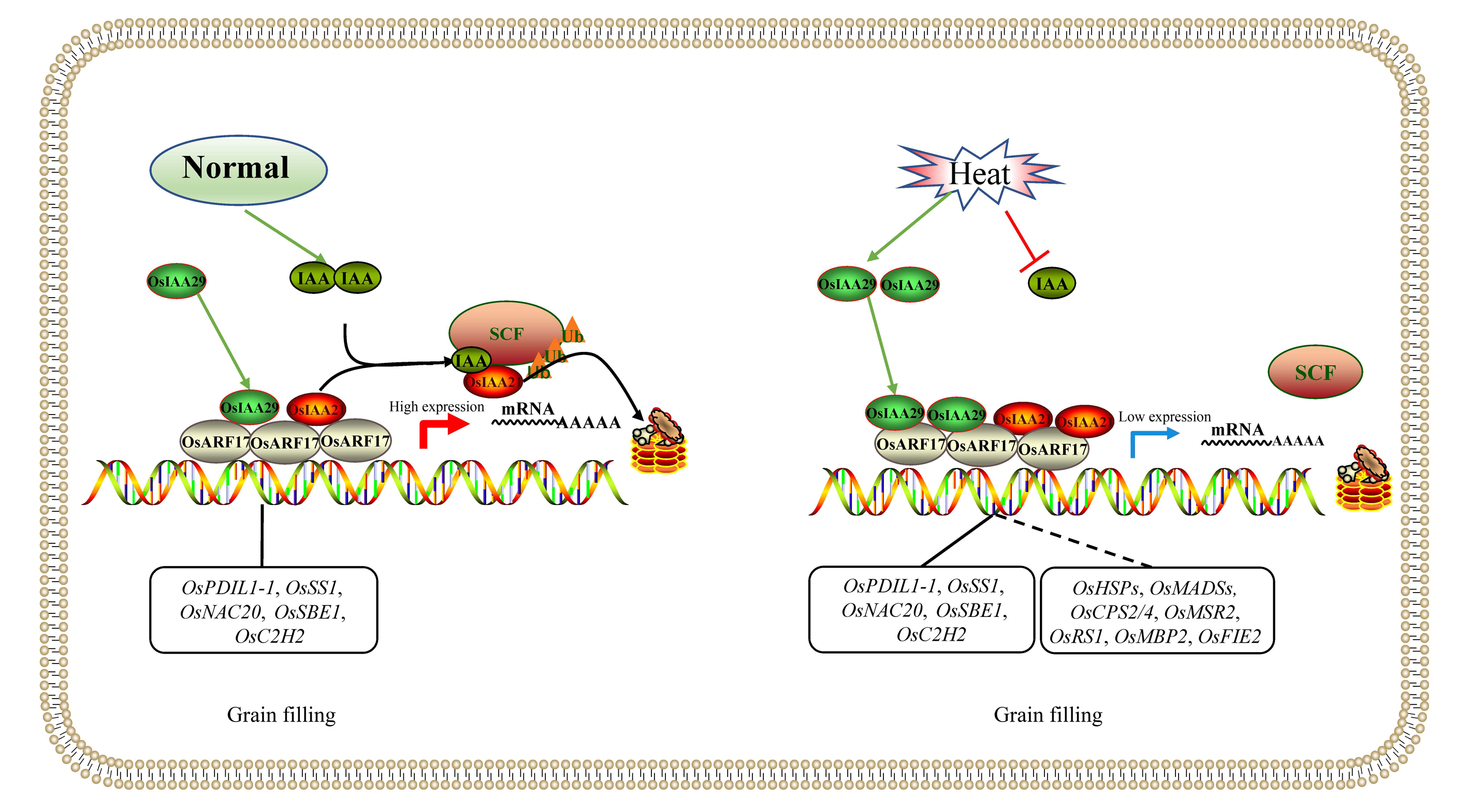


**Figure S7. Schematic diagram of the OsIAA29 response heat stress in rice endosperm.** OsIAA29 plays upstream regulatory roles in endosperm development, and the accumulation of storage substances. When the seeds under heat stress conditions, the auxin level is low, the OsIAA29 protein is induced by heat stress, which leads to a high level of OsIAA29 protein, and OsARF17 are compete by OsIAA21 and OsIAA29 protein. When the seeds under normal conditions, the auxin level is normal, both OsIAA29 and OsIAA21 are degraded by a 26S proteasome-dependent mechanism, but OsIAA29 protein is slowly degraded, and the transcription activity of OsARF17 was released.

| **Table S1. Primers for functional analysis of *OsIAA29*** | | | | | | |  |
| --- | --- | --- | --- | --- | --- | --- | --- |
| Name | Purpose | | | Forward primer (5'–3') | Reverse primer (5'–3') | |  |
| qIAA29 | | IAA29 | AGATGGAGATGGAGCTGCTG | | | TCAGTCGAAGCTTGGGTCAT | |
| qARF17 | | ARF17 | ATAACCCAAGAGCAAGCCCT | | | TGATTCATCCCAGCCAACCT | |
| qC2H2 | | C2H2 | CGGAGATGATCGACTTGAACAG | | | TACACGAGCAGATTAAGCAGAT | |
| *qOSNAC024* | | *NAC24* | CATGAGTACCGTCTCACAAACA | | | GTTGAAGATCTTGCATAGCACC | |
| *qOsSBEI* | | *SBEI* | TCAGCAGCAGATCAGGAACA | | | TGGTAGCCCCTATCTCCTGT | |
| q-OsISA | | *ISA* | ATGCCCCTTTCCTTGTGAGA | | | TGTTCACCTCAGCCCATCTT | |
| q-C2H2 | | *C2H2* | CTACCAACAACCAGCGGC | | | TCAAGTCGATCATCTCCGGC | |
| q-OsARID | | *ARID* | AGAGGACAAGGCAAGGTACC | | | ACTGCTGCTGTATCCTTGGT | |
| qPDIL1-1 | | PDIL1-1 | GCAGCACCCCTAAAGTTGTT | | | TCCCTCAGCCCAAAGTACTG | |
| *qOSNAC020* | | *NAC020* | GATCTCAACAAGTGCGAGCC | | | CCTGCCCTTGTAGAAGACGA | |
| ARF17-HIS | | Pull-down | AGCAAATGGGTCGCGGATCCATGAGGCTTTCGTCGTCGTCC | | | TGTCGACGGAGCTCGAATTCTCAGAATTCAACTGAGCCGACAGATG | |
| IAA29-GST | | Pull-down | ATCTGGTTCCGCGTGGATCCATGAAGGATAGGAATGCTTCTGCT | | | TCGAGTCGACCCGGGAATTCGTCGAAGCTTGGGTCATCGTTG | |
| IAA21-MYC | | Pull-down | CTGAAGAGGATCTGGGATCCATGGCGCCGCCACAGGAGC | | | TCGAGTCGACCCGGGAATTCTCAGTTCCGATTTTTGCTCTTGTC | |
| PMYFP-ARF17 | | localization | GCAGATCTATCGATTCTAGAATGAGGCTTTCGTCGTCGTCC | | | TTGCTCACCATGGCTCTAGAGAATTCAACTGAGCCGACAGATGC | |
| PMYFP-IAA29 | | localization | GCAGATCTATCGATTCTAGAATGAAGGATAGGAATGCTTCTGCT | | | TTGCTCACCATGGCTCTAGAGTCGAAGCTTGGGTCATCGTTG | |
| ARF17-CR | | CRISPR | CCCCTTGTTTCGTTGCCTGCAGTGTTTTAGAGCTAGAAATAGC | | | ACTGCAGGCAACGAAACAAGGGGGCCACGGATCATCTGCACAA | |
| IAA29-CR | | CRISPR | GGGGGTACCATGAAGGATAGGAATGCTTCTG | | | GGGGGATCCGTCGAAGCTTGGGTCATCGT | |
| PVC-IAA29 | | BIFC | GGCGCGCCACTAGTGGATCCATGAAGGATAGGAATGCTTCTGCT | | | ACAGTACTATCGATGGATCCTCAGTCGAAGCTTGGGTCATC | |
| PVN-ARF17 | | BIFC | GGCGCGCCACTAGTGGATCCATGAGGCTTTCGTCGTCGTCC | | | ACAGTACTATCGATGGATCCTCAGAATTCAACTGAGCCGACAGATG | |
| NONE-IAA29 | | Luciferase | TCTCTAGAACTAGTGGATCCATGAAGGATAGGAATGCTTCTGCT | | | ATAAGCTTGATATCGAATTCTCAGTCGAAGCTTGGGTCATC | |
| 35S-IAA29-LUC | | Luciferase | GGAGAGGTCACGCTGGATCCATGAAGGATAGGAATGCTTCTGCT | | | TTGTTGGTAATTGTGGATCCGTCGAAGCTTGGGTCATCGTTG | |
| 35S-IAA21-LUC | | Luciferase | GGAGAGGTCACGCTGGATCCATGGCGCCGCCACAGGAGC | | | TTGTTGGTAATTGTGGATCCGTTCCGATTTTTGCTCTTGTCAGT | |
| DBD-ARF17 | | Luciferase | TCTAGAACTAGTGGATCCATGAGGCTTTCGTCGTCGTCC | | | ATAAGCTTGATATCGAATTCTCAGAATTCAACTGAGCCGACAGATG | |
| IAA29-AD | | Y2H | CCATGGAGGCCAGTGATGAAGGATAGGAATGCTTCTGCTG | | | TGCCCACCCGGGTGGTCAGTCGAAGCTTGGGTCATCGT | |
| IAA29-BD | | Y2H | TATGGCCATGGAGGCCGATGAAGGATAGGAATGCTTCTGCTG | | | CGACGGATCCCCGGGTCAGTCGAAGCTTGGGTCATCGT | |
| ARF17-AD | | Y2H | CCATGGAGGCCAGTGATGAGGCTTTCGTCGTCGTCC | | | AGCTCGAGCTCGATGGAATTCAACTGAGCCGACAGATGCAATTC | |
| *PBRIDGE-ARF17* | | Y3H | TGACTGTATCGCCGGAATTCATGAGGCTTTCGTCGTCGTCC | | | GGCTGCAGGTCGACGGATCCGAATTCAACTGAGCCGACAGATGCAATTC | |
| *PBRIDGE-IAA29* | | Y3H | CCGCATTAGCCCGAAGATCTATGAAGGATAGGAATGCTTCTGCT | | | GGAGATCAGCCCGAAGATCTTCAGTCGAAGCTTGGGTCATC | |
| IAA21-BD | | Y2H | GGCCGAATTCCCGGGGATCCATGGCGCCGCCACAGGAGC | | | TAGTTATGCGGCCGCTGCAGGTTCCGATTTTTGCTCTTGTC | |
| IAA29-CR | | CRISPR | GGGGGTACCATGAAGGATAGGAATGCTTCTG | | | GGGGGATCCGTCGAAGCTTGGGTCATCGT | |
| IAA29-Ri | | RNAi | GGGACTAGTGGTACCCTGCTGACGATGGCGTTGCT | | | GGGGAGCTCGGATCCCACTGAGATGGCACAAGGAC | |
| 1301-ARF17-FLAG | | OE | TAGCCGGTACCCGGGGATCCATGAGGCTTTCGTCGTCGTCC | | | TGATCTTTGTAATCGGATCCTCAGAATTCAACTGAGCCGACAGATG | |
| 1301-IAA29-GFP | | OE | ATAGCCGGTACCCGGGGATCATGAAGGATAGGAATGCTTCTGCTG | | | GCCCTTGCTCACCATGGATCCTCAGTCGAAGCTTGGGTCATC | |
| 2181-ARF17-GUS | | GUS | GCGCTGAAGCTTGGCTGCAGGGTGGTGTTTGGATCCAGAGACT | | | GACTGACCACCCGGGGATCCTACTTCTAACTTGTTCTTGGGGTTTTAAC | |
| 2181-IAA29-GUS | | GUS | TGAAGCTTGGCTGCAATTAAGGGGTTGTGTTGCACTTTTTAAATG | | | GACTGACCACCCGGGCCTACTCCTTATCAATCTGATTTGGTCTCTG | |
| *ChIP-NAC24-1* | | ChIP-qPCR | GCCTTTACAATACTGTAGAG | | | CTACCATCTTATAGGCATTT | |
| *ChIP-NAC24-2* | | ChIP-qPCR | GATGGTAGAGGCATTTTC | | | GGTTAGTGCATTGAAGAT | |
| *ChIP-NAC24-3* | | ChIP-qPCR | CAATGCACTAACCAACTC | | | GCGCCTGATATGTAAAGG | |
| *ChIP-NAC24-4* | | ChIP-qPCR | ATCAGGCGCATTTTACAG | | | TACTCACACAGTACAACA | |
| *ChIP-C2H2-1* | | ChIP-qPCR | GCATGCGTTGATGGCCGCC | | | GACACACATGTTGGTGGTCT | |
| *ChIP-C2H2-2* | | ChIP-qPCR | CCAACATGTGTGTCCCTT | | | GTCACGTAAGAGAAAAGTCC | |
| *ChIP-C2H2-3* | | ChIP-qPCR | CTCTTACGTGACATTAGGC | | | CCGATAGTTATTTTCGGA | |
| *ChIP-ARID-1* | | ChIP-qPCR | GATAGATGAGATTCTACATATC | | | GCACAACACAACAAAACAAGG | |
| *ChIP-ARID-2* | | ChIP-qPCR | GTTGTGTTGTGCAACTCA | | | GGATAGAGATGAGATGATC | |
| *CHIP-ARID-3* | | ChIP-qPCR | CATCTCATCTCTATCCATGT | | | GGTGTTGACGTTGGATAT | |
| *ChIP-*NAC20-1 | | ChIP-qPCR | CCATGTGTTTCTATATGCAG | | | CACAAGCAACACCGATCC | |
| *ChIP-*NAC20-2 | | ChIP-qPCR | TCGGTGTTGCTTGTGAGC | | | GATGTATCGCGTGTTTGGCT | |
| *ChIP-*NAC20-3 | | ChIP-qPCR | AACACGCGATACATCATAACG | | | CAATTGTGAATGCTTATGCCC | |
| *ChIP-*ISA-1 | | ChIP-qPCR | AGCCGAGCTCCGCCGCCTCGA | | | TGTGGGCTCTCTAGATGGCCT | |
| *ChIP-*ISA-2 | | ChIP-qPCR | CATCTAGAGAGCCCACAA | | | GCCATGGTGGCACTGGGA | |
| *ChIP-*ISA-3 | | ChIP-qPCR | AGTGCCACCATGGCGCCCAA | | | ATCCCTTCGGTTTTTGCGT | |
| *ChIP-*SS1-1 | | ChIP-qPCR | TCGAAATTCGAGTTGAAG | | | GTAATTAGTTTCCAGTATTACC | |
| *ChIP-*SS1-2 | | ChIP-qPCR | CTAATTACGTAAAAGGAAAA | | | CACGCAGTTGGTTGCCACATT | |
| *ChIP-*SS1-3 | | ChIP-qPCR | GCAACCAACTGCGTGAAA | | | GCGAGTAATAATACAAAACTCC | |
| *ChIP-*SS1-4 | | ChIP-qPCR | TTGTATTATTACTCGCAC | | | TGCTAGTGGCGGAGAGAA | |
| *ChIP-*BE1-1 | | ChIP-qPCR | TGGACACCTGCCACGTGG | | | TCTCATTACCTTTCGCTT | |
| *ChIP-*BE1-2 | | ChIP-qPCR | GGTAATGAGATTTCTATGC | | | CCACTGCTTTAGCTATCAA | |
| *ChIP-*BE1-3 | | ChIP-qPCR | GATAGCTAAAGCAGTGGGG | | | GTCTCGTGAATTAGCTTT | |
| *ChIP-*BE1-4 | | ChIP-qPCR | GACTACTTACAAAACTAA | | | TCTTGGACTAATTTGCGAG | |
| *ChIP-*PDIL1-1 | | ChIP-qPCR | TCGTTTGCGTTGCGGCAG | | | CTCGTGATCGACAAGTTTA | |
| *ChIP-*PDIL1-2 | | ChIP-qPCR | ACTTGTCGATCACGAGAATG | | | ACGTTTGATGTGATATGGTG | |
| *ChIP-*PDIL1-3 | | ChIP-qPCR | CACCATATCACATCAAACGT | | | GATTAGGGCTATTATCTATC | |
| *ChIP-*PDIL1-4 | | ChIP-qPCR | ATTCGATGCCTTAGGTCC | | | ACTAAGTAAATCCTTCACGG | |
| *ChIP-*PDIL1-5 | | ChIP-qPCR | CCGTGAAGGATTTACTTAGT | | | CGATTGGTTACACGAGTT | |
| *ChIP-*PDIL1-6 | | ChIP-qPCR | AACTCGTGTAACCAATCG | | | CTTGGTCCGTTACGCGCG | |
| *ChIP-*PDIL1-7 | | ChIP-qPCR | CGCGCGTAACGGACCAAG | | | CGAGAAGCGCTTGGAGGTT | |
| PROBE1 | | EMSA | CGTCAATGTATCGTGTAGGGTCCGATGTCTCCGAGTTCTACTTGGAAACCGCCTGACCT | | | AGGTCAGGCGGTTTCCAAGTAGAACTCGGAGACATCGGACCCTACACGATACATTGACG | |
| PROBE2 | | EMSA | TCTCTCGAAACAATAAAATGTGTCTCTTATCTATATGACCGTGAACGTGAACACATTCT | | | AGAATGTGTTCACGTTCACGGTCATATAGATAAGAGACACATTTTATTGTTTCGAGAGA | |

**Table S2. Detection of potential off-target sites for the sgRNAs**

| **Site** | **Chromosome** | **Position** | **Guide-PAM sequence** | **Mismatch numbers** | **Editing** |
| --- | --- | --- | --- | --- | --- |
| On target (*OsIAA29*) | 11 | 6356885 | ATCACCATCAAGATTTGTGA**AGG** | 0 | Yes |
| (Off target 1) LOC_Os11g11420 | 11 | 6348542 | GTCACCATCAAGGTTTGTGA**AGG** | 2 | No |
| (Off targe 2)  LOC_Os06g35870 | 6 | 20930824 | TTCTCCAGCAAGATTGGTGA**AGG** | 4 | No |

**Table S3 Agronomic traits of *osiaa29* under normal and high temperature in 2018**

| Lines |  | Normal temperature | |  |  | High temperature | |
| --- | --- | --- | --- | --- | --- | --- | --- |
|  | Shrunken grain rate (%) | 1000-grain weight (g) | Chalkiness |  | Shrunken grain rate (%) | 1000-grain weight (g) | Chalkiness |
|  |  |  | rate (%) |  |  |  | rate (%) |
| ZH11 | 4.82±0.77 | 24.50±0.12 | 8.50±3.50 |  | 19.27±9.63 | 24.48±0.25 | 19.83±3.52 |
| *osiaa29-1* | 8.46±2.20 | 23.00±0.05 | 19.75±1.25* |  | 98.90±1.55** | 20.52±0.22** | 94.00±2.75** |
| *osiaa29-2* | 5.20±0.00 | 23.10±0.15 | 30.25±0.25** |  | 80.03±16.83** | 21.39±0.13** | 54.00±3.27** |

Data are presented as means standard error (SE) of five biological replicates. *P*-values were calculated using two-tailed *t*-test. **P* < 0.05, ***P* < 0.01.

**Table S4 Agronomic traits of WT and *OsIAA29-*RNAi lines under high temperature in 2019**

| Lines | No. of floret per panicles | Seed set rate  (%) | 1000 grain weight  (g) | Shrunken seeds rate (%) |
| --- | --- | --- | --- | --- |
| WT | 148±14 | 87.46±5.30 | 25.23±0.83 | 1±0.81 |
| Ri29-1 | 146±13 | 64.09±12.15** | 24.92±0.30* | 10.91±1.35** |
| Ri29-2 | 151±18 | 63.39±7.17** | 24.37±0.16** | 12.36±2.4** |

Data are presented as means ± standard error (SE) of three biological replicates. **P* < 0.05, and ***P* < 0.01 using Student’s *t*-test.

**Table S5 Agronomic traits of *osarf17* under normal and high temperature in 2021**

| Lines | Normal temperature | |  | High temperature | |  |
| --- | --- | --- | --- | --- | --- | --- |
|  | Shrunken grain rate (%) | 1000-grain weight (g) |  | Shrunken grain rate (%) | 1000-grain weight (g) | |
|  |  |  |  |  |  |  |
| ZH11 | 1.32±0.27 | 27.54±0.39 |  | 4.01±1.92 | 25.57±0.42 | |
| *osarf17* | 2.00±0.57 | 27.12±0.17 |  | 15.76±1.80** | 25.07±0.63* | |

Data are presented as means standard error (SE) of five biological replicates. *P*-values were calculated using two-tailed *t*-test. **P* < 0.05, ***P* < 0.01.

**Table S6 qRT-PCR analysis of selected genes in *osiaa29-1***

| Name | Gene product | Locus No. | qRT-PCR Change Fold |
| --- | --- | --- | --- |
| *OsSSIVa* | Starch synthase IVa | LOC_Os01g52260 | 4.33 |
| *OsSSIVb* | Starch synthase IVb | LOC_Os05g45720 | 2.77 |
| *OsBEIIa* | Starch branching enzyme IIa | LOC_Os04g33460 | 1.46 |
| *OsBEIIb* | Starch branching enzyme IIb | LOC_Os02g32660 | 0.48 |
| *OsSSI* | Starch synthase I | LOC_Os06g06560 | 0.24 |
| *OsBEI* | Starch branching enzyme I | LOC_Os06g51084 | 0.44 |
| *OsAP2/EREBP* | AP2 domain containing protein, expressed | LOC_Os08g41030 | 4.32 |
| *OsbZIP34* | transcription factor, putative, expressed | LOC_Os03g59460 | 2.14 |
| *OsbZIP35* | transcription factor, putative, expressed | LOC_Os04g10260 | 1.31 |
| *OsC2H2* | ZOS5-09 - C2H2 zinc finger protein, expressed | LOC_Os05g38600 | 0.47 |
| *OsC3H* | zinc finger C-x8-C-x5-C-x3-H type family protein | LOC_Os01g07930 | 3.15 |
| *OsEIL4* | ethylene-insensitive 3, putative, expressed | LOC_Os08g39830 | 1.38 |
| *Osmyb4* | MYB family transcription factor, putative, expressed | LOC_Os01g50110 | 12.74 |
| *OsMYB* | MYB family transcription factor, putative, expressed | LOC_Os01g63680 | 0.97 |
| *OsMYB* | MYB family transcription factor, putative, expressed | LOC_Os01g74590 | 3.09 |
| *Osmyb3* | myb-like DNA-binding domain containing protein | LOC_Os03g29614 | 0.39 |
| *OsMYB* | MYB family transcription factor, putative, expressed | LOC_Os07g31470 | 2.10 |
| *OsNAC20* | no apical meristem protein, putative, expressed | LOC_Os01g01470 | 0.02 |
| *OsNAC26* | no apical meristem protein, putative, expressed | LOC_Os01g29840 | 3.00 |
| *OsNAC23* | no apical meristem protein, putative, expressed | LOC_Os02g12310 | 1.81 |
| *OsNAC024* | no apical meristem protein, putative, expressed | LOC_Os05g34310 | 0.58 |
| *OsNAC* | no apical meristem protein, putative, expressed | LOC_Os11g31360 | 1.08 |
| *OsNAC* | no apical meristem protein, putative, expressed | LOC_Os11g31380 | 1.05 |
| *OsOrphans* | response regulator receiver domain containing protein | LOC_Os04g28120 | 2.19 |
| *OsYC11* | histone-like transcription factor and archaeal histone | LOC_Os10g11580 | 2.24 |
| *OsPHD* | PHD-finger domain containing protein, putative | LOC_Os02g09910 | 2.96 |
| *OsARID1* | high mobility group, putative, expressed | LOC_Os02g27060 | 0.63 |
| *OsSigma70-like* | RNA polymerase sigma factor, putative, expressed | LOC_Os03g16430 | 0.7 |
| *OsHB* | homeobox domain containing protein, expressed | LOC_Os06g39906 | 0.87 |
| *OsRPBF* | dof zinc finger domain containing protein, putative | LOC_Os02g15350 | 0.35 |
| *OsISA2* | Starch debranching enzyme: Isoamylase II | LOC_Os05g32710 | 0.19 |
| *OsRISBZ1* | bZIP transcription factor domain containing protein | LOC_Os07g08420 | 0.53 |
| *OsbHLH144* | helix-loop-helix DNA-binding domain containing protein | LOC_Os04g35010 | 2.32 |
| *OsPHOL* | Starch phosphorylase L | LOC_Os03g55090 | 2.91 |
| *OsAGPL2* | ADP-glucose pyrophosphorylase large subunit 2 | LOC_Os01g44220 | 0.23 |
| *OsPDIL1-1* | OsPDIL1-1 protein disulfide isomerase PDIL1-1, expressed | LOC_Os11g09280 | 0.36 |
| *OsGRAS* | GRAS family transcription factor domain containing protein | LOC_Os05g49930 | 1.44 |
| OsHAP5K | histone-like transcription factor and archaeal histone | LOC_Os05g23910 | 0.00 |
| OsFl3 | zinc-binding protein, putative, expressed | LOC_Os01g33350 | 0.00 |

qRT-PCR change fold is the relative expression of detected gene in *osiaa29* compared with WT.
